# Crowdsourcing to expand HIV testing among men who have sex with men in China: A closed cohort stepped wedge cluster randomized controlled trial

**DOI:** 10.1371/journal.pmed.1002645

**Published:** 2018-08-28

**Authors:** Weiming Tang, Chongyi Wei, Bolin Cao, Dan Wu, Katherine T. Li, Haidong Lu, Wei Ma, Dianmin Kang, Haochu Li, Meizhen Liao, Katie R. Mollan, Michael G. Hudgens, Chuncheng Liu, Wenting Huang, Aifeng Liu, Ye Zhang, M. Kumi Smith, Kate M. Mitchell, Jason J. Ong, Hongyun Fu, Peter Vickerman, Ligang Yang, Cheng Wang, Heping Zheng, Bin Yang, Joseph D. Tucker

**Affiliations:** 1 University of North Carolina Project-China, Guangzhou, China; 2 Social Entrepreneurship to Spur Health (SESH) Global, Guangzhou, China; 3 Institute for Global Health and Infectious Diseases, School of Medicine, University of North Carolina at Chapel Hill, Chapel Hill, North Carolina, United States of America; 4 Dermatology Hospital, Southern Medical University, Guangzhou, China; 5 School of Public Health, Southern Medical University, Guangzhou, China; 6 Social and Behavioral Health Sciences, School of Public Health, Rutgers University, Piscataway, New Jersey, United States of America; 7 School of Media and Communication, Shenzhen University, Shenzhen, China; 8 Weill Cornell Medical College, New York, New York, United States of America; 9 Gillings School of Global Public Health, University of North Carolina, Chapel Hill, North Carolina, United States of America; 10 Shandong University School of Public Health, Jinan, China; 11 Shandong Center for Disease Control and Prevention, Jinan, China; 12 Department of Sociology, University of California San Diego, La Jolla, California, United States of America; 13 Department of Epidemiology and Community Health, University of Minnesota Twin Cities, Minneapolis, Minnesota, United States of America; 14 Department of Infectious Disease Epidemiology, Imperial College London, London, United Kingdom; 15 Faculty of Infectious and Tropical Diseases, London School of Hygiene and Tropical Medicine, London, United Kingdom; 16 Division of Community Health and Research, Eastern Virginia Medical School, Norfolk, Virginia, United States of America; 17 School of Social and Community Medicine, University of Bristol, Bristol, United Kingdom; University of California, San Francisco, UNITED STATES

## Abstract

**Background:**

HIV testing rates are suboptimal among at-risk men. Crowdsourcing may be a useful tool for designing innovative, community-based HIV testing strategies to increase HIV testing. The purpose of this study was to use a stepped wedge cluster randomized controlled trial (RCT) to evaluate the effect of a crowdsourced HIV intervention on HIV testing uptake among men who have sex with men (MSM) in eight Chinese cities.

**Methods and findings:**

An HIV testing intervention was developed through a national image contest, a regional strategy designathon, and local message contests. The final intervention included a multimedia HIV testing campaign, an online HIV testing service, and local testing promotion campaigns tailored for MSM. This intervention was evaluated using a closed cohort stepped wedge cluster RCT in eight Chinese cities (Guangzhou, Shenzhen, Zhuhai, and Jiangmen in Guangdong province; Jinan, Qingdao, Yantai, and Jining in Shandong province) from August 2016 to August 2017. MSM were recruited through Blued, a social networking mobile application for MSM, from July 29 to August 21 of 2016. The primary outcome was self-reported HIV testing in the past 3 months. Secondary outcomes included HIV self-testing, facility-based HIV testing, condom use, and syphilis testing. Generalized linear mixed models (GLMMs) were used to analyze primary and secondary outcomes. We enrolled a total of 1,381 MSM. Most were ≤30 years old (82%), unmarried (86%), and had a college degree or higher (65%). The proportion of individuals receiving an HIV test during the intervention periods within a city was 8.9% (95% confidence interval [CI] 2.2–15.5) greater than during the control periods. In addition, the intention-to-treat analysis showed a higher probability of receiving an HIV test during the intervention periods as compared to the control periods (estimated risk ratio [RR] = 1.43, 95% CI 1.19–1.73). The intervention also increased HIV self-testing (RR = 1.89, 95% CI 1.50–2.38). There was no effect on facility-based HIV testing (RR = 1.00, 95% CI 0.79–1.26), condom use (RR = 1.00, 95% CI 0.86–1.17), or syphilis testing (RR = 0.92, 95% CI 0.70–1.21). A total of 48.6% (593/1,219) of participants reported that they received HIV self-testing. Among men who received two HIV tests, 32 individuals seroconverted during the 1-year study period. Study limitations include the use of self-reported HIV testing data among a subset of men and non-completion of the final survey by 23% of participants. Our study population was a young online group in urban China and the relevance of our findings to other populations will require further investigation.

**Conclusions:**

In this setting, crowdsourcing was effective for developing and strengthening community-based HIV testing services for MSM. Crowdsourced interventions may be an important tool for the scale-up of HIV testing services among MSM in low- and middle-income countries (LMIC).

**Trial registration:**

ClinicalTrials.gov NCT02796963

## Abstract 2

In a randomized controlled trial, Joseph Tucker and colleagues study the effect of a crowdsourced intervention on HIV testing among men who have sex with men in China.

## Author summary

**Why was this study done?:**

- HIV testing remains low among key populations, including MSM, especially in low- and middle-income countries.
- Although crowdsourcing has been recommended as a tool for developing public health interventions, few studies have formally evaluated crowdsourcing as a way to promote health in a real-world setting.
- Previous studies on crowdsourcing have suggested that it may be a promising approach to increase HIV testing among MSM in China.

**What did the authors do and find?:**

- We investigated the effectiveness of a crowdsourced intervention in promoting HIV testing among Chinese MSM in eight cities distributed in two provinces.
- We found that the crowdsourced intervention was effective in promoting HIV testing. Compared to the control period, the intervention period showed an 8.9% absolute increase (and a 43% relative increase) in HIV testing.
- The intervention was particularly effective in promoting HIV self-testing.

**What do these findings mean?:**

- In this setting, we found crowdsourcing was a way to develop innovative and effective HIV testing services. Further research is needed to examine this approach in other settings.
- A crowdsourcing approach can help build and sustain community engagement related to HIV testing.
- Programs aiming to expand HIV testing should consider crowdsourcing as a tool to design tailored services.

## Introduction

Approximately 14 million people living with HIV have yet to be tested, compromising the effectiveness of HIV treatment and prevention programs [1]. Testing rates are particularly poor among key populations (e.g., men who have sex with men [MSM], 25%–32%) in low- and middle-income countries (LMIC) [1,2]. Entrenched community norms that marginalize key populations, limited HIV resources, and insufficient community awareness all contribute to low levels of HIV testing around the world, including China [3–5].

In China, HIV infection rates are still increasing among MSM, highlighting the need for innovative HIV testing interventions [6,7]. Recent global literature suggests that community involvement and social media can be important tools in reaching high-risk populations and improving HIV testing rates [8,9]. In China, community engagement has also been shown to be an important predictor of HIV testing [10], and previous community-based interventions have shown promise [11,12].

Crowdsourcing can be a useful tool to develop community-driven HIV testing services in LMIC [13]. It allows a group, including experts and nonexperts, to solve problems and share solutions with the public [13]. Crowdsourcing is a scalable, cost-effective tool to aggregate community wisdom [14]. Crowdsourcing approaches have been used to organize half a million people to help identify viral protein structures [15], deployed laypeople for cardiopulmonary resuscitation in out-of-hospital cardiac arrest [16], and solicited videos that promote HIV testing [14].

However, the potential for crowdsourcing as a tool to develop community health services, such as HIV testing, is uncertain; crowdsourcing for health improvement has focused on developing single components of health campaigns [17], rather than creating a comprehensive service. Pilot trials have suggested that crowdsourcing may be a useful tool for developing images and videos to promote HIV testing [14,18]. Further research is needed to examine whether crowdsourcing can effectively improve HIV testing services among key populations in LMIC.

The purpose of this stepped wedge cluster randomized controlled trial (RCT) was to evaluate a comprehensive crowdsourced intervention to increase HIV testing uptake among MSM in China.

## Methods

### Summary

The study protocol provides a more detailed description of the trial design and analysis plan (S1 Text) [19]. Briefly, we developed an intervention consisting of a multimedia HIV testing campaign, an online HIV testing service, and local testing promotion campaigns tailored for MSM. The intervention was developed using crowdsourcing and consisted of three components: a nationwide open contest call for images and concepts that encourage HIV testing, a 72-hour regional designathon for developing HIV testing strategies [20], and local participatory contests soliciting HIV testing messages. We then conducted a closed cohort stepped wedge cluster RCT in eight Chinese cities to evaluate the impact of this crowdsourced intervention compared to conventional programs routinely provided by local Centers for Disease Control (CDCs) and community-based organizations (CBOs). The intervention was implemented over 12 months. We followed standard guidelines for reporting stepped wedge cluster RCTs (S1 CONSORT Checklist) [21].

### Intervention development

**Fig 1:**
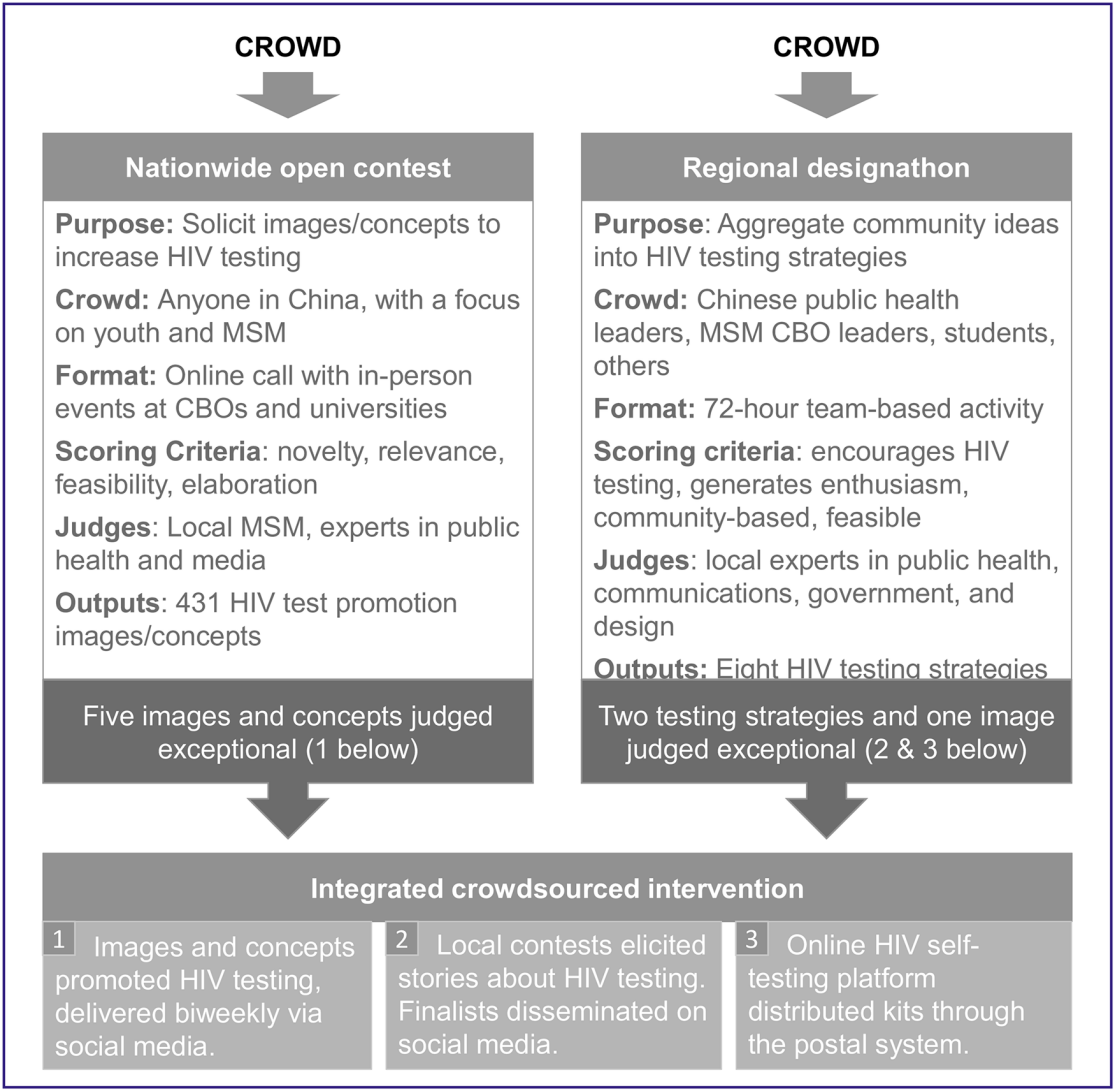
Steps in intervention development. Details are provided in S2 Text. *Exceptional defined by ranking after scores from three independent judges. CBO, community-based organization; MSM, men who have sex with men.

The steps for intervention development are shown in Fig 1 and further explained in S2 Text. Three participatory activities shaped the content and structure of the intervention: a nationwide open contest, a regional strategy designathon, and local participatory contests.

#### Nationwide open contest

The nationwide open contest solicited concepts and images promoting HIV testing for 6 weeks, which was conducted from March to April of 2016. We promoted the contest through social media and in-person community events with CBO partners. Three social media platforms promoted the contest: WeChat, an instant messaging system similar to Facebook and Twitter; Weibo, a Chinese microblogging platform; and QQ, an instant messaging software. The four largest cities in Guangdong and Shandong Provinces (Guangzhou, Shenzhen, Qingdao, and Jinan) were selected to implement additional in-person community events to promote the open contest. In-person events included classroom presentations led by contest organizers, feedback sessions for potential contest entries, and announcements during events. Entries were evaluated by local MSM and experts using a 10-point scale (1 = worst; 10 = best). The top 40 entries (out of 431) were used as brainstorming materials for the regional designathon. Five images from this contest judged as exceptional were ultimately used in the final intervention as promotional materials.

#### Regional strategy designathon

The regional designathon was conducted in May of 2016. It was a 3-day, hackathon-like event, during which teams of five each developed an HIV testing service strategy [20]. The strategies consisted of concepts, images, and implementation plans. There were eight teams, one from each of the eight participating cities (Guangzhou, Shenzhen, Zhuhai, and Jiangmen in Guangdong province; Jinan, Qingdao, Yantai, and Jining in Shandong province). Each team consisted of one CDC worker for the local city, one MSM CBO leader from the local city, and three other participants selected from a nationwide applicant pool. Teams were provided with the top 40 entries from the national crowdsourcing contest. Teams then had 72 hours to develop an HIV testing service strategy. The strategies were evaluated by a steering committee (five members) consisting of local professionals in public health, communications, civil society, and design. Judging criteria included (1) potential to encourage HIV testing, (2) potential to generate enthusiasm, (3) community-based, and (4) feasible in the local context [20]. Judges evaluated each strategy and provided feedback to teams. The two best-scoring strategies were an HIV self-testing platform delivered by social media and a series of local HIV testing story contests (S2 Text). These two strategies were incorporated into the final intervention.

#### Integrated intervention components

**Fig 2:**
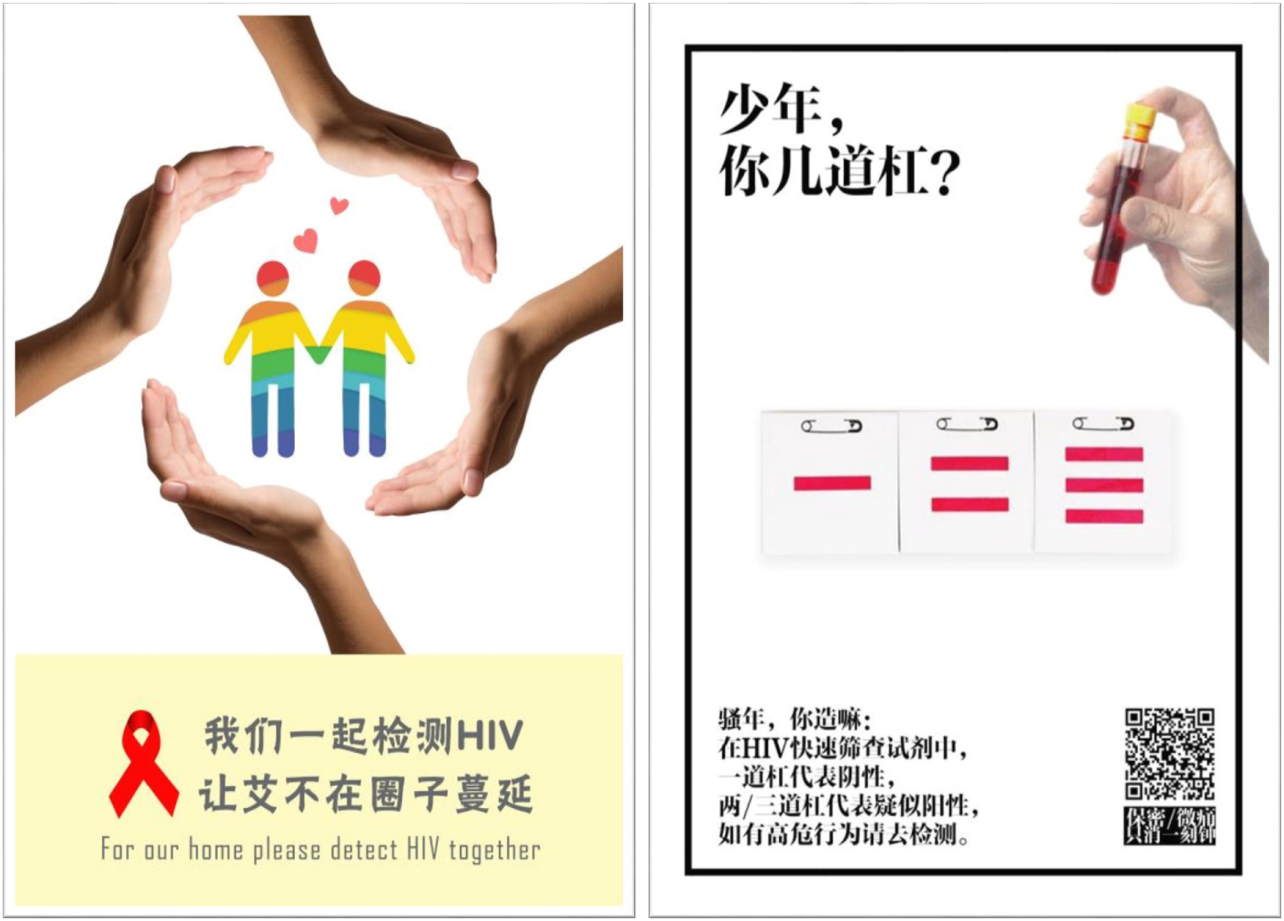
Two of the six crowdsourced HIV promotion images used in the intervention package. The six images were delivered biweekly during the 3-month intervention period via WeChat. Left text: “Let’s test for HIV together. Stop HIV from spreading in our community.” Right text: “Son, what’s your rank? HIV test: one line means negative; two or three lines means suspected positive. Please go and get HIV tested”.

The final intervention integrated exceptional images from the national contest (Fig 2) and the two top-ranked strategies that resulted from the designathon—an HIV self-testing platform delivered by social media and a series of local HIV testing story contests. The exceptional images were disseminated by WeChat biweekly during the intervention period (S2 Text). The HIV self-testing platform was built in WeChat, and men who were interested could provide their address to receive one free oral HIV self-test kit in the mail. Men could also return a photo of the test results through WeChat. The local story contests were co-organized by respective local CDCs and MSM CBOs in the eight cities and aimed to promote continuing community engagement and HIV testing by soliciting stories of HIV testing from local individuals (S3 Text, three stories selected from the story contests). Exceptional stories from the story contests were disseminated through social media channels of local CBOs, and those who submitted exceptional entries were awarded prizes.

### Study setting, recruitment, and randomization

We selected four cities each from Guangdong province (Guangzhou, Shenzhen, Zhuhai, and Jiangmen) and Shandong province (Jinan, Qingdao, Yantai, and Jining). Each city had existing infrastructure for MSM HIV surveillance led by the local CDC and capacity to deliver new HIV testing services. Participants were eligible if they were born biologically male, age 16 or older, currently living and planning to live in one of the eight cities for 12 months post-enrollment, HIV-negative or unknown HIV status, had not had HIV testing within the past 3 months, had anal sex with a man at least once during their lifetime, and were willing to provide their cell phone numbers for follow-up. We recruited participants through China’s largest MSM social networking mobile phone application (app), Blued, by sending a survey invitation to registered users in the eight selected cities.

We randomly assigned the order of intervention for each of the four cities in Guangdong province and Shandong province, then paired the cities by order of intervention (S1 Table). Prior to receiving the intervention, cities were considered to be in the control state. We initiated the intervention for each pair at 3-month intervals, and each pair of cities received the intervention for 3 consecutive months. In total, we collected data at baseline followed by four data collection points over 12 months.

### Follow-up

**Fig 3:**
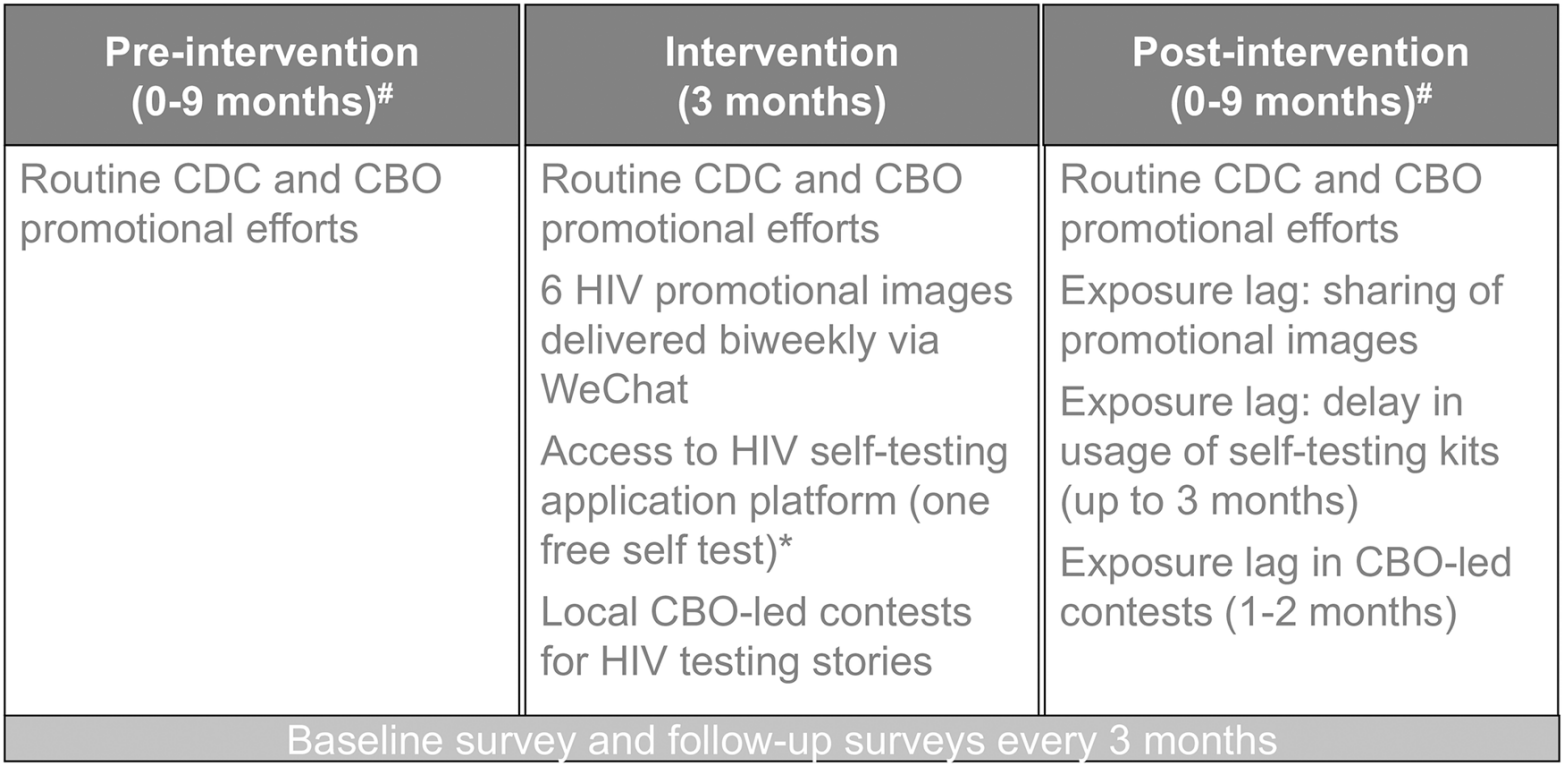
Conditions during the control, intervention, and post-intervention phases. *Self-testing platform for Group 1 was delayed until the post-intervention period. ^#^Length of these periods varied by randomization group. CBO, community-based organization; CDC, Center for Disease Control.

After electronically signing an informed consent form, all participants were asked to fill out a survey at baseline, and at every 3 months thereafter. Any participant who returned a photo of a positive test result was counseled to seek out confirmatory testing from their local CDC or CBO and was no longer eligible for subsequent follow-up surveys. Conditions of the control state, intervention, and post-intervention period are shown in Fig 3.

### Sample size

To determine the necessary sample size for recruitment, we assumed that a crowdsourced intervention would be more effective than a conventional method for promoting HIV testing [14]. We assumed an HIV testing rate of 25% during the control period and 35% during the intervention period. These assumptions were made based on existing levels of HIV testing under conventional HIV testing promotion and pilot data evaluating crowdsourced HIV testing videos in China [14]. With eight clusters (cities), four intervention time lines, a coefficient of variation of 0.4, a 0.05 significance level, 90% power, and 30% loss to follow-up, we planned to recruit at least 1,040 men from the eight cities (130 from each city).

### Measures

The primary outcome of this study was the proportion of participants who tested for HIV over the previous 3 months. This was assessed based on self-reported data from each follow-up survey. We also measured the following secondary outcomes: facility-based HIV testing; HIV self-testing; syphilis testing; condomless sex; using WeChat, Weibo, QQ, or mobile phone applications to give/receive information about HIV testing; anticipated HIV stigma; HIV testing social norms; HIV testing self-efficacy; and community engagement in sexual health. We adapted validated scales to measure anticipated HIV stigma [22], HIV testing social norms [23], HIV testing self-efficacy [24], and increase in community engagement in sexual health during the follow-up period (as compared to baseline) [10]. The primary and secondary outcomes were measured at baseline and in each of the four follow-up surveys at 3, 6, 9, and 12 months after baseline. We also measured incident HIV testing during the study period, defined as a participant’s first HIV test during the study period, as well as incident HIV testing among participants who had never tested for HIV at baseline.

### Statistical analysis

Descriptive analysis was used to summarize the demographic characteristics and HIV testing proportions. To investigate the effect of the intervention, we evaluated the difference in probability of HIV testing in the control period and in the intervention period (including the post-intervention period). We applied intention-to-treat analysis utilizing generalized linear mixed models (GLMMs) to evaluate the primary and secondary study outcomes. Intervention status and time indicators allowing for piecewise secular trends were considered fixed effects, while sites and individual participants with multiple measurements across the four follow-ups were considered random effects [25]. We treated all intervention periods and post-intervention periods in the same manner. The estimated intervention effect sizes (risk ratio [RR] for binary outcomes, and mean difference for continuous outcomes) were each reported with a 95% confidence interval (CI) and *p*-value. We encountered non-convergence problems when a log-binomial GLMM was used to estimate relative risk. Thus, we employed a log Poisson GLMM model [26].

Sensitivity analyses were conducted to evaluate a per-protocol effect and a city/cluster-level effect of the intervention on HIV testing proportion in the past 3 months. The per-protocol analysis only included participants who reported viewing the intervention materials. For the city-level intervention effect, we used the HIV-testing proportion of each city across the four follow-ups as the outcome, and fit a normal linear mixed model that accounted for the random effect of sites to estimate the difference in HIV testing proportions. An intention-to-treat sensitivity analysis was conducted to evaluate the effectiveness of the intervention while adjusting for the province in the model. According to our prespecified analysis plan, we tested interactions with age (>30 years old versus ≤30 years old) and for cities with in-person community activities during the intervention development phase (Jinan, Qingdao, Guangzhou, and Shenzhen). We also used multiple imputations to examine the intervention effect. Using multiple imputations by chained equations and 30 imputed datasets, missing HIV testing data were imputed at each follow-up period using a logit model that included baseline variables (age, marital status, province, and income) and intervention status during the respective follow-up period. A log Poisson GLMM was fit to estimate relative risk. Rubin’s rules were employed to pool the parameter estimates using the MIANALYZE Procedure in SAS [27]. All data analyses were completed using SAS 9.4 (Cary, NC).

### Ethical statement

Ethical approval was obtained from the institutional review committees at the Guangdong Provincial Center for Skin Diseases and STI Control (Guangzhou, China), Shandong University (Jinan, China), University of North Carolina at Chapel Hill (Chapel Hill, NC), and the University of California, San Francisco (San Francisco, CA). The trial is registered with ClinicalTrials.gov (NCT02796963).

## Results

### Study participants

Participants were recruited from July 29, 2016, and followed until August 21, 2017. Overall, the study link was clicked 39,764 times. Of these, 16,193 withdrew from the survey prior to reading the consent form and 21,187 people did not meet eligibility requirements. Among the remaining, 1,003 did not sign the informed consent form. (S1 Fig)

**Fig 4:**
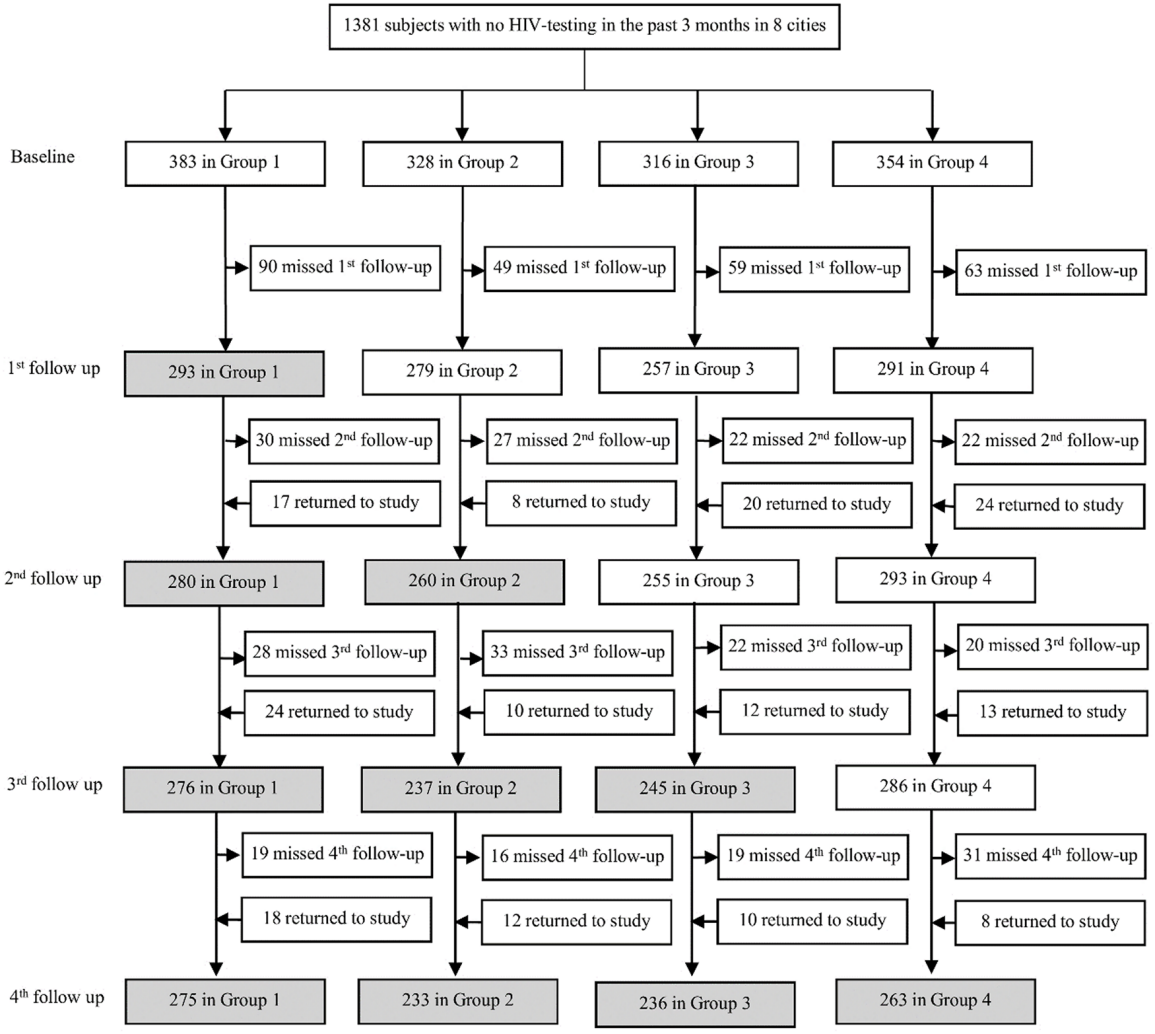
Trial profile. The intervention was implemented in a closed cohort stepped wedge design. Shaded cells represent intervention and post-intervention periods and white cells represent the control period. Each group consists of two cities: one each from Guangdong and Shandong provinces. Group 1—Guangzhou and Yantai; Group 2—Jiangmen and Jinan; Group 3—Zhuhai and Qingdao; Group 4—Shenzhen and Jining. Surveys were distributed at each follow-up.

A total of 1,381 participants were enrolled. Of these, 203, 139, 134, and 203 were recruited from Guangzhou, Jiangmen, Zhuhai, and Shenzhen, respectively, in Guangdong province, while 180, 189, 182, and 151 were recruited from Yantai, Jinan, Qingdao, and Jining, respectively, in Shandong province (Fig 4, S1 Table).

### Randomization and follow-up

Based on the predetermined randomization schedule, 383 participants from Guangzhou and Yantai were assigned to the first intervention group (Group 1), 328 participants from Jiangmen and Jinan were assigned to the second group (Group 2), 316 participants from Zhuhai and Qingdao were assigned to the third group (Group 3), and 354 participants from Shenzhen and Jining were assigned to the fourth group (Group 4) (Fig 4, S1 Table).

Among the 1,313 HIV-negative or status unknown participants after the third follow-up, 306 did not finish our last survey, with a loss-to-follow-up rate of 23% (306/1,313, Fig 4). Loss-to-follow-up rates were similar between the four intervention groups. Characteristics of participants lost to follow-up differed in age and income from participants who completed the last follow-up (S2 Table).

A total of 1,219 participants completed at least one follow-up survey. Participants who missed one follow-up survey but rejoined the study at a subsequent survey date were assumed not to have tested during the missed follow-up period.

### Baseline characteristics of the participants

Demographic characteristics of the participants were similar across the four groups (Table 1). The majority of participants were 30 years old or younger (82%), had never married (86%), had a college degree or higher (65%), had an annual income below US$9,500 (74%), and had disclosed their sexual orientation (65%). Most participants (95%) identified as male and the rest identified as transgender. Seventy-one percent reported their sexual orientation as “tongxinglian,” gay. In addition, 73% of the participants reported that they had engaged in condomless sex in the past 3 months and 5% tested for syphilis in the past 3 months. At baseline, 57% of participants had never tested for HIV.

**Table 1:** Baseline characteristics of study participants (stratified by intervention group*) in a crowdsourcing stepped wedge cluster randomized controlled trial in China in 2016–2017 (*N* = 1,381).

Characteristics	Group 1 (*n* = 383)	Group 2 (*n* = 328)	Group 3 (*n* = 316)	Group 4 (*n* = 354)	Total (*n* = 1,381)
Gender					
Male	364 (95%)	314 (96%)	301 (95%)	334 (94%)	1,313 (95%)
Transgender^†^	19 (5%)	14 (4%)	15 (5%)	20 (6%)	68 (5%)
Age, years					
16–20	65 (17%)	78 (24%)	81 (26%)	87 (25%)	311 (23%)
21–25	146 (38%)	118 (36%)	94 (30%)	111 (31%)	469 (34%)
26–30	100 (26%)	80 (24%)	80 (25%)	95 (27%)	355 (26%)
>30	72 (19%)	52 (16%)	61 (19%)	61 (17%)	246 (18%)
Marital status					
Never married	337 (88%)	290 (88%)	268 (85%)	299 (84%)	1,194 (86%)
Married	46 (12%)	38 (11%)	48 (15%)	55 (16%)	187 (13%)
Annual income, US$					
<3,000	75 (20%)	82 (25%)	64 (20%)	64 (18%)	285 (21%)
3,000–6,000	75 (20%)	86 (26%)	71 (22%)	67 (19%)	299 (22%)
6,001–9,500	133 (35%)	88 (27%)	93 (29%)	120 (34%)	434 (31%)
9,501–15,000	65 (17%)	50 (15%)	52 (16%)	67 (19%)	234 (17%)
≥15,001	35 (9%)	22 (7%)	36 (11%)	36 (10%)	129 (9%)
Highest education					
High school or below	132 (34%)	106 (32%)	118 (37%)	127 (36%)	483 (35%)
College or beyond	251 (65%)	222 (67%)	198 (63%)	227 (65%)	898 (65%)
Sexual orientation					
Gay	296 (77%)	225 (69%)	230 (73%)	229 (65%)	980 (71%)
Bisexual	87 (23%)	103 (31%)	86 (27%)	125 (35%)	401 (29%)
Disclosure of sexual orientation^¶^					
Disclosed to others	262 (68%)	205 (63%)	209 (66%)	222 (63%)	898 (65%)
Not disclosed to others	121 (32%)	123 (38%)	107 (34%)	132 (37%)	483 (35%)
Condomless sex^‡^					
No	101 (26%)	92 (28%)	85 (27%)	98 (28%)	376 (27%)
Yes	282 (74%)	236 (72%)	231 (73%)	256 (72%)	1,005 (73%)
Syphilis testing^‡^					
No	360 (94%)	317 (97%)	301 (95%)	335 (95%)	1,313 (95%)
Yes	23 (6%)	11 (3%)	15 (5%)	19 (5%)	68 (5%)
Ever tested for HIV					
No	219 (57%)	181 (55%)	173 (55%)	217 (61%)	790 (57%)
Yes	164 (43%)	147 (45%)	143 (45%)	137 (39%)	592 (43%)

Data are *n* (%).

*Group 1: Guangzhou, Yantai; Group 2: Jiangmen, Jinan; Group 3: Zhuhai, Qingdao; Group 4: Shenzhen, Jining.

^†^Born biologically male and now identifies as female or transgender.

^¶^Has told anyone (except sexual partners) about sexuality or sexual history with men.

^‡^In the past 3 months.

### The proportion of participants who tested for HIV

Overall, the proportion testing for HIV in the last 3 months increased after the intervention and this trend was maintained during the post-intervention periods (Table 2). Similar results were also found for each study city (S1 Table). The number of incident testers in each follow-up period and the number of incident testers who had never tested for HIV prior to baseline are shown in S3 Table. Generally, incidence of HIV testing was high during the intervention period.

**Table 2:** HIV testing rates by intervention group over four follow-up periods among Chinese MSM, 2016–2017 (*N* = 1,219).

Group	Enrollment, *n*	HIV testing proportion in the past three months, percent (participants tested/total participants)*			
		1st follow-up	2nd follow-up	3rd follow-up	4th follow-up
Group 1	383	19.1 (56/293)	35.4 (99/280)	25.4 (70/276)	32.0 (88/275)
Group 2	328	19.7 (55/279)	32.7 (85/260)	29.1 (69/237)	36.5 (85/233)
Group 3	316	19.8 (51/257)	23.9 (61/255)	49.8 (122/245)	39.4 (93/236)
Group 4	354	21.3 (62/291)	28.3 (83/293)	29.0 (83/286)	48.7 (128/263)

Follow-ups took place at 3-month intervals. Group 1 represents Guangzhou and Yantai; Group 2, Jiangmen and Jinan; Group 3, Zhuhai and Qingdao; Group 4, Shenzhen and Jining. Intervention periods are shown in dark gray, post-intervention periods in light gray. A total of 755 unique individuals out of 1,219 participants who filled out at least one of the four follow-up surveys tested over the intervention period (62%) tested during the follow-up period.

*We included 1,219 participants who filled out at least one of the four follow up surveys in this analysis.

Abbreviation: MSM, men who have sex with men.

The proportion of individuals receiving an HIV test within a city was 8.9% (95% CI 2.2–15.5) greater during the intervention periods (Table 3). In addition, results from the intention-to-treat analysis showed that the probability of an individual HIV testing during the intervention periods (including post-intervention periods) was higher than in the control periods (estimated RR = 1.43, 95% CI 1.19–1.73) (Table 3). Multiple imputation analysis produced a similar result. In the per-protocol analysis, the estimated effect size was larger (RR = 1.49, 95% CI 1.21–1.83).

**Table 3:** Effect of crowdsourced intervention on uptake of HIV testing among Chinese MSM, 2016–2017: Generalized linear mixed models (*N* = 1,219).

Effect	Estimate (95% CI)^£^	*p*-value	ICC by city
Risk ratio			
HIV testing in the past three months (individual level)			
Intervention effect assuming fixed secular trend	1.43 (1.19, 1.73)	<0.001	0.016
Per-protocol effect*	1.49 (1.21, 1.83)	<0.001	0.020
Intervention effect adjusted for province^¶^	1.47 (1.21, 1.78)	<0.001	0.011
Intervention effect adjusted for age, marital status, and income	1.43 (1.18, 1.73)	<0.001	0.016
Intervention effect using multiple imputation	1.43 (1.17, 1.69)	<0.001	---
By age group		0.52 (Interaction)	0.017
Age ≤30	1.41 (1.16, 1.72)		
Age >30	1.57 (1.12, 2.21)		
By in-person community activities		0.27 (Interaction)	0.020
Cities with in-person community activities^1^	1.56 (1.24, 1.96)		
Cities without in-person community activities^2^	1.35 (1.06, 1.73)		
Risk (probability) difference, percent			
City-level HIV testing in the past three months			
Weighted by sample size for each city	8.9 (2.2, 15.5)	0.01	---

*Assuming fixed secular trend across clusters.

^¶^Adjusted for the province (Guangdong and Shandong Provinces).

^1^Jinan, Qingdao, Guangzhou, Shenzhen.

^2^Jining, Yantai, Jiangmen, Zhuhai.

^**£**^We included 1,219 participants who filled out at least one of the four follow-up surveys in this analysis.

Abbreviations: CI, confidence interval; ICC, intraclass correlation coefficient; MSM, men who have sex with men.

The model with interaction term suggested that the intervention effect was similar among MSM who were 16 to 30 years old (RR = 1.41, 95% CI 1.16–1.72) compared to MSM over 30 years old (RR = 1.57, 95% CI 1.12–2.21, interaction test *p* = 0.52). In addition, the intervention had a similar effect in cities with in-person community activities (RR = 1.56, 95% CI 1.24–1.96) compared to cities that did not have in-person activities (RR = 1.35, 95% CI 1.06–1.73, interaction test *p* = 0.27) (Table 3).

### Cumulative HIV testing uptake

Out of 1,219 participants who completed at least one follow-up survey, 755 (62%) unique participants self-reported that they tested for HIV at any point during the study period. Of these, 642 (85%) tested during the intervention and post-intervention periods (S3 Table). Of the 699 participants who had never tested for HIV at baseline, 390 (56%) tested at any point during the study period (S3 Table). A total of 395 participants reported testing only once during the follow-up period, whereas 360 participants tested during more than one follow-up period (211 people tested twice, 107 people tested three times, and 42 people tested four times [S4 Table]).

A total of 593 participants (49% of 1,219 participants who completed at least one follow-up) received HIV self-testing. Of these, 442 (75%) used our self-testing platform, and 132 (30%) returned their self-test results via WeChat. Among these individuals, seven (5%) confirmed results were positive. We found that 93.9% of self-reported results were consistent with the returned photo of results (S5 Table).

By the end of the study period, 99 participants (50 from Guangdong province; 49 from Shandong province) reported a positive HIV test result. The cumulative HIV prevalence among those who tested for HIV was 13.1%. Of these, 32 individuals seroconverted during the 1-year study period. Seroconversion was defined has having an initial negative HIV test and then a subsequent positive HIV test.

### Secondary outcomes

Table 4 reports the results for secondary outcomes using an intention-to-treat approach. Our intervention increased HIV self-testing (RR = 1.89, 95% CI 1.50–2.38, S6 Table; S4 Text lists further statistical considerations). There was no difference in facility-based HIV testing between the intervention and control periods (RR = 1.00, 95% CI 0.79–1.26). The crowdsourced intervention also did not improve condom use, syphilis testing, or anticipated HIV stigma (Table 4).

**Table 4:** Effect of crowdsourced intervention on secondary outcomes among Chinese MSM, 2016–2017: Generalized linear mixed models (*N* = 1,219).

Secondary outcomes^¶^	Estimated risk ratio (95% CI)*	*p*-value	ICC by city
HIV self-testing	1.89 (1.50, 2.38)	<0.001	0.028
HIV facility-based testing	1.00 (0.79, 1.26)	0.99	0.002
Condom use	1.00 (0.86, 1.17)	0.96	0.007
Syphilis testing	0.92 (0.70, 1.21)	0.55	0.005
Using Weibo to give/receive information^#^	0.95 (0.77, 1.19)	0.66	0.010
Using WeChat to give/receive information^#^	1.18 (0.51, 2.75)	0.24	<0.001
Using QQ to give/receive information^#^	0.88 (0.71, 1.09)	0.25	0.030
Using gay mobile phone apps to give/receive information^#^	0.95 (0.35, 2.56)	0.62	<0.001
Increased community engagement^£^	0.97 (0.44, 2.12)	0.70	<0.001
	Estimate (95% CI)*	*p*-value	
	Mean difference		
Anticipated HIV stigma	−0.027 (−0.064, 0.010)	0.15	0.006
HIV testing social norms	−0.010 (−0.041, 0.020)	0.51	0.002
HIV testing self-efficacy	−0.008 (−0.039, 0.023)	0.62	<0.001

*Adjusted for secular time trend as a fixed effect across clusters.

^#^Using WeChat, Weibo, QQ, or Blued to give or receive information about HIV testing, excluding receipt of intervention materials.

^**¶**^We included 1,219 participants who filled out at least one of the four follow-up surveys in this analysis.

^£^Defined as whether the cumulative community engagement score increased, by comparing to the baseline.

Abbreviations: CI, confidence interval; ICC, intraclass correlation coefficient; MSM, men who have sex with men.

## Discussion

HIV testing is an essential first step in the HIV care continuum. Although HIV testing is a major global health priority, in key populations large numbers of people remain untested [28]. We recruited MSM from eight Chinese cities and followed individuals longitudinally for 12 months to evaluate the effect of an intervention developed through crowdsourcing on promoting HIV testing. We found that the crowdsourced intervention was effective in promoting HIV testing compared to the control period, showing an 8.9% absolute increase (and a 43% relative increase) in HIV testing during the intervention period. The intervention was particularly useful in promoting HIV self-testing. Our study extends previous research on crowdsourcing by using it to develop a comprehensive HIV testing service, evaluating its effectiveness in a pragmatic trial, and assessing the long-term effect of the intervention [17]. In contrast with our study, nearly all of the limited crowdsourcing health research studies have been observational to date [17].

Within our study cohort, 62% of participants self-reported that they received HIV testing at least once during the study period, and 56% of previously untested MSM received HIV testing. This is consistent with the literature on global community-based HIV testing promotion [14,28,29] but breaks new ground in formally evaluating crowdsourcing as an approach for developing HIV testing services to identify untested individuals. The HIV prevalence we observed was higher than that reported among studies from MSM in Shandong province [30,31], suggesting a higher burden of HIV among previously untested MSM populations. However, it also suggests that community-based, crowdsourced interventions are capable of reaching these populations. Given the relatively low cost of crowdsourcing [14], these types of approaches may be useful in a number of LMIC settings.

The World Health Organization now recommends HIV self-testing [32]. While there is evidence suggesting that HIV self-testing can increase HIV testing among MSM [33–37], studies have not previously measured longitudinal effects in China or described community input. In our study, 49% of participants underwent HIV self-testing, 75% of whom used our self-testing platform, further supporting the acceptability of HIV self-testing. Our study also showed that the crowdsourced intervention increased HIV self-testing but not facility-based testing, highlighting the need for further research to enhance linkage to HIV prevention and other services.

Our data indicated that the intervention was effective among both young and older MSM. Young MSM were heavily invested in all of the participatory activities that shaped the design and implementation of the intervention. Given the high rates of WeChat/Weibo use among youth, we speculate that crowdsourcing may be particularly effective among young MSM who use social media. Considering that our intervention engaged substantial youth in each city to provide feedback about HIV services [17,20], crowdsourcing may help to develop more youth-friendly HIV services.

Our study has several research and policy implications. It suggests that crowdsourcing could be used to design tailored HIV services, providing direction for subsequent crowdsourcing research. Also, crowdsourcing provides an inclusive, effective way to solicit community input; hence, it might be used to inform health policy [38,39]. Finally, when planning HIV interventions for MSM, researchers and policy makers should consider social media interventions to expand dissemination [9].

Our study has several limitations. First, most HIV testing data were based on self-report. Nevertheless, previous studies have demonstrated that HIV test self-report reliably correlates with operational HIV testing data [40,41]. Within the sample of participants who returned images of their self-testing kit results, the vast majority of returned results was consistent with the self-reported results (S5 Table). Second, 23% of the non-seroconverted men did not finish our last survey, a relatively large loss to follow-up. Characteristics of participants lost to follow-up differed from participants who completed the last follow-up in age and income (S2 Table). However, our findings were robust when adjusted for age and income, and also when using multiple imputation analysis. Third, this study was limited in its generalizability. The RCT only included MSM recruited online. However, a previous study suggested that findings from online MSM studies in China are comparable to national MSM data [42]. Additionally, this study recruited mostly young men from urban China. Further research is needed among groups of different ages, cultures, and locations. Fourth, the implementation of HIV self-testing was delayed in two cities because of logistical problems. This may explain why HIV testing rates were lower in the earlier groups, which would bias our effect estimates towards the null, suggesting that reported results are even more conservative than the true effect. Fifth, we did not collect data on linkage to care. Linkage to care is a key component for HIV testing. Previous work has shown that CBOs can adopt innovative methods to promote linkage to care, which can be incorporated into future interventions [43]. Finally, there may have been contamination between the intervention and control periods, especially among participants of the crowdsourcing contest and designathon who may have viewed intervention materials in advance. We did not collect information on whether men participated in contests used to develop the intervention. Because it would be impossible to determine if control groups had inadvertently seen the intervention without exposing them to the intervention, we did not collect information on potential spillover effects. However, given that the intraclass correlation for participants within each city was low, we anticipate that the impact of the spillover would also be small.

In summary, crowdsourcing can help spur the development of new HIV testing services. Our data demonstrate that a crowdsourcing approach effectively increased HIV testing in Chinese cities, especially HIV self-testing among MSM. While the crowdsourcing approach was implemented across cities, each city’s local contests helped shape and contextualize testing messages for local communities. Crowdsourcing approaches may be an important tool for localizing and differentiating HIV services.

## Supporting information

S1 Text Protocol. (DOCX)

S2 Text Crowdsourcing intervention implementation. (DOCX)

S3 Text Three stories selected from story contests in eight Chinese cities. (DOCX)

S4 Text Statistical considerations. (DOCX)

S1 Fig Trial recruitment results. (TIF)

S1 CONSORT Checklist Cluster trials checklist. (DOCX)

S1 Data Deidentified survey data. (XLSX)

S1 Table HIV testing rate by city among Chinese MSM in China, 2016–2017 (*N* = 1,219). MSM, men who have sex with men.
(DOCX)

S2 Table Baseline characteristics of study participants stratified by completion of the last Survey in the crowdsourcing stepped wedge cluster randomized controlled trial in China in 2016–2017 (*N* = 1,381). (DOCX)

S3 Table Incident HIV testers during each follow-up period. (DOCX)

S4 Table HIV testing frequency over four follow-up periods among MSM who tested for HIV (*N* = 755). MSM, men who have sex with men.
(DOCX)

S5 Table Concordance between self-reported testing results and HIV self-testing kit results among participants who used our HIV self-testing platform, 2016–2017 (*n* = 132). (DOCX)

S6 Table HIV self-testing rates by intervention group over four follow-up periods among Chinese MSM, 2016–2017 (*N* = 1,219). MSM, men who have sex with men.
(DOCX)

## Abbreviations

CBO: community-based organization
CDC: Centers for Disease Control
CI: confidence interval
GLMM: generalized linear mixed model
LMIC: low- and middle-income countries
MSM: men who have sex with men
RCT: randomized controlled trial
RR: risk ratio
