# Supplementary material for: Crowdsourcing to expand HIV testing among men who have sex with men in China: A closed cohort stepped wedge cluster randomized controlled trial
Source: PLoS Med. 2018 Aug 28;15(8):e1002645. doi: 10.1371/journal.pmed.1002645 (PMC6112627; doi:10.1371/journal.pmed.1002645)
Supplement: S1 Text — (DOCX) [file pmed.1002645.s001.docx]

# S1 Text. Protocol

**Crowdsourcing to Promote HIV Testing: A Pragmatic Stepped Wedge Randomized Controlled Trial to Evaluate Promoting HIV Testing in China**

**Study Protocol**

**28 April 2017**

**Version 1.8**

**STUDY TEAM**

*Partner Institutions:* Guangdong Provincial Center for Skin Diseases and STI Control, University of North Carolina at Chapel Hill, University of California San Francisco, London School of Hygiene and Tropical Medicine, University of Bristol, Shandong University, Shandong Provincial CDC, Guangdong Provincial CDC, SESH Global

PIs: Joseph D. Tucker, Chongyi Wei

Survey development: Weiming Tang, Songyuan Tang, Chuncheng Liu, Haochu Li

Statisticians: David Glidden, Chongyi Wei, Weiming Tang, Katie Mollan, Michael Hudgens

MSM recruitment: Zihuang Chen, Chuncheng Liu, Larry Han

Social media: Lisa Hightow-Weidman, Chongyi Wei, Kevin Fenton, Adam Saffer, Bolin Cao

Modelling: Peter Vickerman, Kate Mitchell, Fern Terris-Prestholt, Kumi Smith, Jason

Study design: Kumi Smith, Rosanna Peeling, Barry Bayus, Weiming Tang

Guangzhou Team: Bin Yang, Shujie Huang, Weiming Tang, Songyuan Tang, Chuncheng Liu, Wei Zhang, Jessica Mao, Yilu Qin, Rong Fu, Bolin Cao, Ligang Yang, Wang Cheng, Joseph Tucker, Rong Fu

Jinan Team: Haochu Li, Wei Ma, Meizhen Liao, Dianmin Kang, Chongyi Wei

Oversight: Bin Yang, Joseph Tucker

External Advisory Board: Paul Volberding, George Rutherford, William Wong, Jinkou Zhao

*Funding support:* National Institutes of Health (NIAID 1R01AI114310-01), UNC-South China STD Research Training Center (FIC 1D43TW009532-01), UNC Center for AIDS Research (NIAID 5P30AI050410), UCSF Center for AIDS Research (NIAID P30 AI027763), NIMH (R00MH093201), UJMT Fogarty Fellowship (FIC R25TW0093), and SESH Global ([www.seshglobal.org](http://www.seshglobal.org)). Administrative assistance from the Guangdong Provincial Center for Skin Diseases and STI Control. UNC Chapel Hill, and UNC Project-China in Guangzhou, China.

*Addresses:*

| Adam Saffer | UNC School of Media and Journalism, Carroll Hall, CB 3365, Chapel Hill, NC 27599 |
| --- | --- |
| Barry Bayus | McColl 4526, CB #3490, Chapel Hill, NC |
| Chongyi Wei | UCSF School of Medicine, Epidemiology & Biostatistics, 550 16th. Street, San Francisco CA 94158 |
| David Glidden | Box 0560 , UCSF, San Francisco, CA 94143-0560 |
| Fern Terris-Prestholt | 202 London School of Hygiene and Tropical Medicine, 15-17 Tavistock Place, London, WC1H 9SH, UK |
| Guangzhou Team^1^ | University of North Carolina Chapel Hill Project-China, No. 2 Lujing Road, Guangzhou, China, 510095 |
| Jason Ong | Monash University, Wellington Rd & Blackburn Rd, Clayton VIC 3800, Australia |
| Jinan Team^2^ | School of Public Health, Shandong University, Baotuquan Campus, 44 West Wenhua Road, Jinan, China, 250012 |
| Jinkou Zhao | Nanjing Medical University, 140 Hanzhong Rd, Gulou, Nanjing, Jiangsu, China, 210029 |
| Joseph D. Tucker | University of North Carolina Chapel Hill Project-China, No. 2 Lujing Road, Guangzhou, China, 510095 |
| Kate Mitchell | UG3, Norfolk Place, St Mary's Campus, Imperial College London, London, UK |
| Katie Mollan | UNC Center for AIDS Research, Lineberger Cancer Center, Chapel Hill, NC 27599 |
| Kevin Fenton | Public Health England, Wellington House, 133-155 Waterloo Road, London, UK |
| Lisa Hightow-Weidman | Division of Infectious Diseases, CB# 7030, Bioinformatics Building, 130 Mason Farm Road, 2nd Floor, Chapel Hill, NC |
| Michael Hudgens | 3107C McGavran-Greenberg Hall, CB #7420, Chapel Hill, NC 27599 |
| Paul Volberding  George Rutherford | UCSF School of Medicine, Epidemiology & Biostatistics, 550 16th. Street, San Francisco CA 94158 |
| Peter Vickerman | Office OF21, Oakfield House, Oakfield Grove, Clifton BS8 2BN, UK |
| Rosanna Peeling | 305b London School of Hygiene and Tropical Medicine, Keppel Street, London, WC1E 7HT, UK |
| William Wong | 3/F., 161 Main Street,  Ap Lei Chau Clinic, Ap Lei Chau, Hong Kong |
| Zihuang Chen | No. 2-B-028, Ping Guo She Qu, Bai Zi Wan Lu, Chaoyang, Beijing, China |

^1^Guangzhou team includes the following individuals: Weiming Tang, Songyuan Tang, Chuncheng Liu, Larry Han, Bolin Cao, Kumi Smith, Bin Yang, Shujie Huang, Wei Zhang, Jessica Mao, Yilu Qin, Ligang Yang, Wang Cheng, Rong Fu, Tiange Zhang

^2^Jinan team includes the following individuals: Haochu Li, Wei Ma, Meizhen Liao, Dianmin Kang

Table of Contents

[1 List of Abbreviations 5](#_Toc495947898)

[2 Summary 6](#_Toc495947899)

[3 Introduction 8](#_Toc495947901)

[3.1 Background 8](#_Toc495947902)

[3.2 Specific Aims and Hypotheses 10](#_Toc495947907)

[4 Trial Design 10](#_Toc495947908)

[4.1 Type of Trial 10](#_Toc495947909)

[4.2 Rationale for Trial Design 11](#_Toc495947910)

[4.3 Randomization and Stratification 11](#_Toc495947911)

[5 Trial Population and Measures 12](#_Toc495947912)

[5.1 Eligibility 12](#_Toc495947913)

[5.2 Recruitment 12](#_Toc495947914)

[5.2.1 Online Recruitment 12](#_Toc495947915)

[5.2.2 CDC Data Collection 13](#_Toc495947916)

[5.3 Measures 13](#_Toc495947917)

[6 Intervention 13](#_Toc495947918)

[6.1 Intervention Development 13](#_Toc495947919)

[6.1.1 Crowdsourcing contest 13](#_Toc495947920)

[6.1.2 Designathon 14](#_Toc495947921)

[6.2 Intervention Implementation 15](#_Toc495947922)

[7 Statistical Considerations 17](#_Toc495947924)

[7.1 Primary Outcomes 17](#_Toc495947925)

[7.2 Secondary Outcomes 17](#_Toc495947926)

[7.3 Sample Size Calculation 17](#_Toc495947927)

[7.4 Analysis Plan 18](#_Toc495947929)

[7.4.1 Primary Outcome Analyses 18](#_Toc495947930)

[7.4.2 Secondary Outcome Analyses 19](#_Toc495947931)

[7.4.3 Sub-Analyses 19](#_Toc495947932)

[8 Ethical considerations 19](#_Toc495947933)

[8.1 Risk Analysis 19](#_Toc495947934)

[8.1.1 Risks to the patient 19](#_Toc495947935)

[8.1.2 Benefits of the study 19](#_Toc495947936)

[8.2 Confidentiality 21](#_Toc495947937)

[8.3 Monitoring procedures 22](#_Toc495947938)

[8.4 Compliance 22](#_Toc495947939)

[8.5 Participant withdrawal 22](#_Toc495947941)

[8.6 Institutional review board 23](#_Toc495947942)

[8.7 Informed consent 23](#_Toc495947943)

[9 Appendix 1. Secondary outcomes measured as part of this RCT. 25](#_Toc495947944)

[10 References 27](#_Toc495947945)

# List of Abbreviations

CBO Community-based organization

CDC Centers for Disease Control and Prevention

CI Confidence interval

GD Guangdong Province

GEE Generalized estimating equations

GLMM Generalized linear mixed models

HIVST HIV self-testing

MSM Men who have sex with men

RCT Randomized controlled trial

SD Shandong Province

SESH Social Entrepreneurship for Sexual Health Group

SMS Short Message Service text message

# Summary

**Purpose**: The purpose of this study is to determine whether crowdsourcing can increase uptake of HIV testing

**Objectives**:

The primary objective of this study is to compare HIV testing uptake resulting from a crowdsourced HIV testing promotion intervention compared to a conventional HIV testing campaigns.

The secondary objectives of this study are to compare the following outcomes between a crowdsourced intervention and a conventional media campaign:

- Syphilis testing uptake
- Incremental cost
- HIV self-testing
- Condom usage and other safe-sex behaviors
- Social media engagement
- Perceptions of stigma

**Design**: This is a pragmatic step-wedged randomized controlled, multi-center trial

Sites: Eight cities in two provinces in China: four from Guangzhou Province (Guangzhou, Jiangmen, Zhuhai, Shenzhen) and four from Shandong Province (Yantai, Jinan, Qingdao, Jining)

**Duration**: 16 months: three months of intervention development, thirteen months of implementation and follow-up

**Study population**: Men who have sex with men located geographically in the eight included cities. Under current assumptions, 1280 participants (160 per city) will be enrolled.

**Study procedures:**

**Intervention development**: First, a nationwide crowdsourcing contest will be held to solicit images and concepts to promote HIV testing and sexual health. Entries will be judged by public health and media professionals, and the winning entry will be selected for implementation. Next, a 72-hour designathon will be held to generate an implementation plan for the winning crowdsourced entry. Each team will consist of one CDC worker and one MSM CBO leader for each of the cities, as well as three participants selected from a nationwide application. A group of judges will evaluate team entries and select design elements to be included in a final, HIV test promotion campaign.

**Implementation**: The final campaign will be implemented using social media, in-person events, and other crowdsourced ideas. We will randomize the eight participating cities to begin implementing the campaign two cities at a time every three months.

Enrollment: We will advertise participation in the trial via banner advertisements on BlueD, China’s largest gay dating mobile app. No names or addresses will be collected

**Data collection:** We will administer an online survey with questions pertaining to the outcomes of interest at four points throughout the trial: once at enrollment, and every three months thereafter.

# Introduction

## Background

In recent years, China has made significant progress against its HIV epidemic. Despite a slow response in the 1980s, the government has since implemented STI surveillance, including comprehensive active surveillance of high-risk groups, including STD clinic attendees, commercial sex workers, injection drug users, long-distance truck drivers, women attending antenatal clinics, and MSM.[^1^](#_ENREF_1) According to surveillance data, the prevalence of nationwide HIV fell to a low of 0.037% in 2014. Among female sex workers, long distance truck drivers, pregnant women, and young students, the prevalence has remained constant or decreased to below 1%. The rate among injection drug users, previously the largest contributor to HIV infections, has fallen from around 7% in 2003-2005 to 3-4% in 2012-2014. However, among men who have sex with men (MSM), the prevalence of HIV has increased from 0.9% in 2003 to 7.7% in 2014, and continues to trend upward.[^2^](#_ENREF_2). One review estimated the prevalence of HIV among MSM to be around 8% in 2015.[^3^](#_ENREF_3)

This is consistent with data on MSM globally, who are 19 times more likely to be have HIV than the general population.[^4^](#_ENREF_4) In 2015, the prevalence of HIV among MSM ranged from 4.3% in South East Asia to 14.9% in the African region[^5^](#_ENREF_5). HIV has risen rapidly among MSM, especially in low and middle-income countries.[^4^](#_ENREF_4)^,^[^6^](#_ENREF_6). Causes for this have been attributed to biological causes, (such as increased risk of infection during receptive anal sex), social causes (such as role reversal larger numbers of casual partners among MSM), as well as structural causes (such as societal stigma and minimal access to HIV services).[^6^](#_ENREF_6)^,^[^7^](#_ENREF_7)

Despite their increased risk, studies in China have shown that few MSM ever test for HIV. Although the Chinese government and community-based organizations provide free HIV and syphilis testing [^8^](#_ENREF_8), stigma, discrimination and fear may prevent MSM from accessing these services [^9^](#_ENREF_9) MSM continue to experience social pressure and stigmatized, and many remain closeted, making them even more difficult to reach [^10^](#_ENREF_10)^,^[^11^](#_ENREF_11). Several systematic reviews report that only half of MSM have ever been tested for HIV. [^12^](#_ENREF_12)^,^[^13^](#_ENREF_13) Current campaign efforts are not adequately reaching Chinese MSM and new approaches are needed. A recent modeling study showed that a four-fold increase in general-population testing rates in China may prevent as many as 42,000 HIV infections and 11,000 deaths over the next 5 years. [^14^](#_ENREF_14)

Recently, public health and sexual health campaigns have begun utilizing social marketing, which is the application of commercial marketing concepts to the development of public health campaigns. [^15^](#_ENREF_15)^,^[^16^](#_ENREF_16) However, social marketing relies heavily on experts, limiting feedback from the communities they intend to reach.

Crowdsourcing may provide a promising strategy for reaching marginalized populations while engaging communities. [^17^](#_ENREF_17)^,^[^18^](#_ENREF_18) Crowdsourcing comprises receiving input from a large group of individuals to solve a problem, the sharing the solution with the community. Crowdsourcing often involves open contests enabled through multi-sectoral partnerships [^19^](#_ENREF_19) [^20^](#_ENREF_20). Originally developed in the private sector to improve commercial products [^20^](#_ENREF_20), crowdsourcing has been successfully used to advance health research in many areas from molecular modeling to public health [^21^](#_ENREF_21).

Crowdsourcing may be particularly suited for engaging young MSM. As crowdsourcing is often enabled by the Internet, young Chinese MSM would be an ideal group of individuals to apply this intervention to because: 1) they have high rates of Internet and social media usage and are technologically savvy; and 2) due to gay stigma, many are not “out” and spend much of their time socializing within online forums. Previous work from our group suggest crowdsourcing may be an effective strategy for expanding HIV services among MSM at a low cost [^22^](#_ENREF_22)^,^[^23^](#_ENREF_23). In the first study, we held an open contest to solicit crowd contributions for an HIV test promotion video. We then evaluated the effectiveness of the winning crowd-sourced contribution against a conventional social marketing video. The study found that 37% of previously never-tested MSM who viewed the crowdsourced video subsequently reported receiving first-time HIV testing within the short term (4 weeks). This was similar to the testing rate observed in the group that viewed a conventional social marketing video, but was far more cost-effective [^22^](#_ENREF_22). The second study evaluated the effectiveness of a crowdsourced condom promotion video against a conventional social marketing video at 3 weeks and 3 months after the intervention. Results demonstrated that the crowdsourced condom promotion video was non-inferior to the social marketing video and cost substantially less [^23^](#_ENREF_23). Additionally, qualitative data from our research group has shown that crowdsourcing contests empower individuals and result in improved engagement from hard-to-reach communities.[^24^](#_ENREF_24)

Despite crowdsourcing’s promise, there is limited research on its potential in designing interventions. In addition, the effectiveness of crowdsourced interventions has not been examined in a range of local settings. Most crowdsourcing studies have been single contests that focused on generating campaign content, such as videos and posters, stopping short of designing an overall implementation plan. Our study aims to sustain crowd contribution through an entire intervention by implementing two serial contests - a content-focused contest followed by a second design-focused contest. Furthermore, our study will expand the understanding of crowdsourcing’s effectiveness through a multi-site design that spans eight cities and assesses long-term effects. Results will reveal insights into qualities that are key to the success of public health interventions.

## Specific Aims and Hypotheses

Specific Aim 1: To compare HIV test uptake associated with a crowdsourced intervention to that associated with conventional HIV test uptake campaigns.

Hypothesis 1: A crowdsourced intervention is superior in eliciting HIV test uptake compared to conventional HIV test uptake campaigns.

Specific Aim 2: To compare secondary outcomes (including incremental cost, condom use, HIV testing social norms, syphilis testing, etc.) of a crowdsourced intervention to those of conventional HIV test uptake campaigns.

Hypothesis 2: A crowdsourced intervention is superior in promoting a range of healthy behaviors and HIV testing social norms.

# Trial Design

## Type of Trial

This study will use an adaptation of the stepped wedge randomized controlled trial (RCT) design (Figure 1). In the stepped wedge RCT, study sites are randomized to begin the intervention at different times so that by the end of the study period all sites have initiated the intervention. A total of eight major metropolitan cities - four from Guangzhou Province (Guangzhou, Jiangmen, Zhuhai, Shenzhen) and four from Shandong Province (Yantai, Jinan, Qingdao, Jining) - will implement the crowdsourced intervention. These cities were chosen based on the following criteria: 1) previous CDC MSM sentinel surveillance site; 2) capacity for campaign implementation; 3) capacity for intervention implementation at community level. Four cities (Guangzhou, Shenzhen in Guangdong Province, Qingdao, and Jinan in Shandong Province) will implement more intensive in-person events to promote engagement during the intervention development phase. Intervention development and implementation are described in detail in later sections.

## Rationale for Trial Design

A number of factors influenced our decision to adopt a pragmatic stepped wedge RCT design. Unlike a tightly controlled explanatory trial, a pragmatic trial evaluates an intervention in a real life context, and is particularly well-suited for service delivery interventions[^25^](#_ENREF_25). Our previous studies showed that a crowdsourcing intervention was non-inferior to conventional social media campaigns [^3^](#_ENREF_3)^,^[^22^](#_ENREF_22). For this trial, we aim to evaluate the effect of a crowdsourced intervention in the real-world context.

Second, we aim to understand the effects of crowdsourced interventions in the range of local settings. A pragmatic design allows us to examine this intervention in eight different cities, which may differ in population, social norms and cultural factors, and local infrastructure. Further, a step-wise design also accounts for the impact of time on the effectiveness of the intervention, for service-delivery interventions which may have a lag time. [^25^](#_ENREF_25).

Finally, our two previous studies demonstrated that crowdsourcing can enhance HIV interventions among MSM.[^3^](#_ENREF_3)^,^[^26^](#_ENREF_26) Given that we will recruit MSM with a known high risk of acquiring HIV infection, withholding our intervention to a subgroup of participants would be ethically questionable. A stepped wedge RCT design addresses this ethical concern by ensuring all participants receive the intervention.

## Randomization and Stratification

The eight cities will be randomized to initiate intervention in groups of two at three-month intervals (Figure 1). The order of intervention implementation at four cities within each province (Guangdong and Shandong Provinces) will be randomized using SAS software. One city in Guangdong Province and one city in Shandong Province will then begin intervention simultaneously, i.e. city-level randomization will be stratified by province. While waiting to initiate the intervention, cities will continue conventional campaigns that are part of the routine activities of local CDC and CBOs.

**Figure 1. Stepped-wedge study design**

| Phase | Prep. | | | Intervention | | | | | | | | | | | |  |
| --- | --- | --- | --- | --- | --- | --- | --- | --- | --- | --- | --- | --- | --- | --- | --- | --- |
| Months | 1 | 2 | 3 | 4 | 5 | 6 | 7 | 8 | 9 | 10 | 11 | 12 | 13 | 14 | 15 | 16 |
| CDC surveillance survey |  |  |  |  |  |  |  |  |  |  |  |  |  |  |  |  |
| Develop intervention and recruit online cohort in 8 cities |  |  |  |  |  |  |  |  |  |  |  |  |  |  |  |  |
| Guangzhou (GD) |  |  |  | * |  |  | * |  |  | * |  |  | * |  |  | * |
| Yantai (SD) |  |  |  | * |  |  | * |  |  | * |  |  | * |  |  | * |
| Jiangmen (GD) |  |  |  | * |  |  | * |  |  | * |  |  | * |  |  | * |
| Jinan (SD) |  |  |  | * |  |  | * |  |  | * |  |  | * |  |  | * |
| Zhuhai (GD) |  |  |  | * |  |  | * |  |  | * |  |  | * |  |  | * |
| Qingdao (SD) |  |  |  | * |  |  | * |  |  | * |  |  | * |  |  | * |
| Shenzhen (GD) |  |  |  | * |  |  | * |  |  | * |  |  | * |  |  | * |
| Jining (SD) |  |  |  | * |  |  | * |  |  | * |  |  | * |  |  | * |

*represents online survey at baseline and every 3 months thereafter. Gray shading represents roll-out of the intervention. 8 city clusters; 5 time periods; randomization stratified by 2 provinces (Guangdong, Shandong)

# Trial Population and Measures

## Eligibility

Eligibility criteria will include: currently living and planning to live in the eight cities for the next 12 months; not living with HIV; no HIV test in the past three months; born biologically male and identify as either male or transgender; had oral or anal sex with men at least once during their lifetime; 16 years and older; willing to provide cell phone number (for follow up and incentive delivery purposes); completed the informed consent document. MSM who meet all other eligibility criteria but received testing in the past three months or living with HIV will be invited to complete a single survey, but not followed over time in the cohort.

## Recruitment

### Online Recruitment

We will build our questionnaire on Sojump Survey Software (Sojump, Shanghai, China). Men will enter the study through website and social media banner/word advertisements, yet only those who lived in the 8 study cities could launch the questionnaire with the survey platform’s IP address restriction function. China’s largest gay app, BlueD, will be used to target recruitment within the eight cities. Eligible men will be invited to join the online cohort. No names or addresses will be collected from participants. In addition to the direct recruitment through websites and social media advertisements, participating individuals will be invited to refer up to three friends from their social networks, and 10 RMB will be given to them as incentive for each eligible participants they successfully invited. All individuals who enroll in the study will receive a 50 RMB (8.50 USD) pre-paid cell phone card for the first follow up and 50 RMB for each subsequent follow up. Those who complete all surveys will be given an opportunity to win an iPad mini. Surveys will be given at baseline and every three months thereafter (Figure 1).

### CDC Data Collection

MSM surveillance sites in each of the eight cities will have additional questions added about viewing SESH images, social media engagement with SESH, contributing to SESH contests, exposure to other ongoing campaigns, HIV/syphilis testing, HIV/syphilis test results. All men who enter surveillance sites in these cities will also be invited to take part in the online cohort. Following informed consent, cell phone numbers will be used to link CDC and online survey data sets.

## Measures

Information on socio-demographics, sexual behaviors, and psychosocial conditions will be collected using standardized online survey tools. Socio-demographic characteristics include participants’ age, highest level of education completed, annual income, marital status, sexual orientation, and sexual orientation disclosure. Behavioral and psychosocial variables include self-reported HIV testing, syphilis testing, HIV self-testing, HIV test-associated stigma, frequency of sex, condom use (condomless sex, sex always with condom, and no sex), HIV testing social norms, HIV testing self-efficacy, community engagement, campaign engagement, MSM empowerment.

# Intervention

## Intervention Development

The intervention will be developed from a nationwide crowdsourcing contest and a designathon (Figure 2). The crowdsourcing contest will generate intervention materials that will later be packaged via the designathon into core elements of an HIV testing campaign. This ensures crowd wisdom is utilized through the entire intervention, from idea generation to campaign implementation.

### Crowdsourcing contest

The first part of the crowdsourcing contest will be an open call for concepts (< 500 characters) or images (photographs, posters, drawings, etc.) promoting HIV testing among young men in China. This open call will be announced on social media platforms nationwide. Social media promotion will include QQ, Weibo, WeChat announcements and short videos explaining the contest from SESH and our community partners in each city (CBOs and student groups interested in HIV testing). Social media will also serve as a channel for announcing prizes, deadlines, and other relevant information. Four cities (Guangzhou, Shenzhen in Guangdong Province, Qingdao, and Jinan in Shandong Province) will implement in-person events in addition to social media promotion. In-person events will include community-based introductions, interactive feedback sessions, and community-driven events (decided by community partners). Multiple incentives, including chances to win an iPad Mini, cash, post cards, etc., will be included to encourage contest participation.

Crowdsourced entries will be evaluated by a crowd panel and an expert panel. The crowd panel consists of MSM from each of the eight cities while the expert panel consists of professionals from CDC, CBOs, and universities in the eight cities. These local panels increase the likelihood that local preferences would be incorporated, which may facilitate later implementation. The quality of crowdsourced ideas will be judged based on four established dimensions: novelty, relevance, feasibility, and elaboration. Judges will consider the four dimensions and score an entry on a 10-point scale. Given that a large number of judges evaluating a relatively small number of entries have been shown to be internally consistent and externally valid, each judge in our contest will evaluate no more than 20 entries. Based on the number of entries, we will ensure enough judges are recruited so that each entry has at least three independent ratings. Following these judging criteria, all entries will first be screened to check for relevance to our contest and plagiarism. Next, 40 concepts and/or images will be identified by the crowd panel and expert panel. Scores from the expert panel will be used to announce contest winners. All 40 concepts and/or images will be recognized as finalist entries and be presented as materials for the designathon. The crowdsourcing contest and judging are planned to span a three-month period.

### Designathon

The designathon will use finalist concepts and/or images to develop core elements of an HIV testing campaign. A designathon is similar to a hackathon,[^27^](#_ENREF_27)^,^[^28^](#_ENREF_28) but focused instead on designing a campaign. Teams are formed with an emphasis on multi-sectoral partnership. Each team consists of one CDC worker and one MSM CBO leader from each of the eight cities as well as three participants selected from a nationwide application. A group of mentors from communication, design, social work, and public health background will also be available for consultation to all teams. Teams will have 72 hours to brainstorm and generate a written intervention plan that incorporates concepts and images from the crowdsourcing contest. A group of judges will evaluate team entries and select design elements to be included in a final, HIV test promotion campaign. Winners will be recognized with awards.

The final campaign will be implemented at both the individual and community levels using social media, in-person events, and other crowdsourced ideas for implementation. After the RCT is complete, we will launch an image bank that allows free access to images/taglines/concepts developed as part of the contest.

## Intervention Implementation

Phased implementation will be carried out in the eight cities following the stepped wedge RCT design (Figure 1). Implementation in each city will be locally adapted based on crowd feedback from the contest and the designathon. The intervention will be implemented at the individual level (via WeChat messages and SMS) and at the community level (via community partners including CDC, CBOs, and social media influencers) (Figure 2).

For individual-level implementation, the campaign content (images and/or concepts) will first be shown to the online cohort at the end of the baseline survey, and then repeated once every two weeks for the next three months. Half of the online cohort will receive the campaign content via WeChat message while the other half will receive the campaign content via SMS text message. For community-level implementation, community partners in each city will facilitate the campaign using crowdsourced implementation ideas generated from the designathon.

**Figure 2. Schematic diagram of intervention development and implementation.**

***
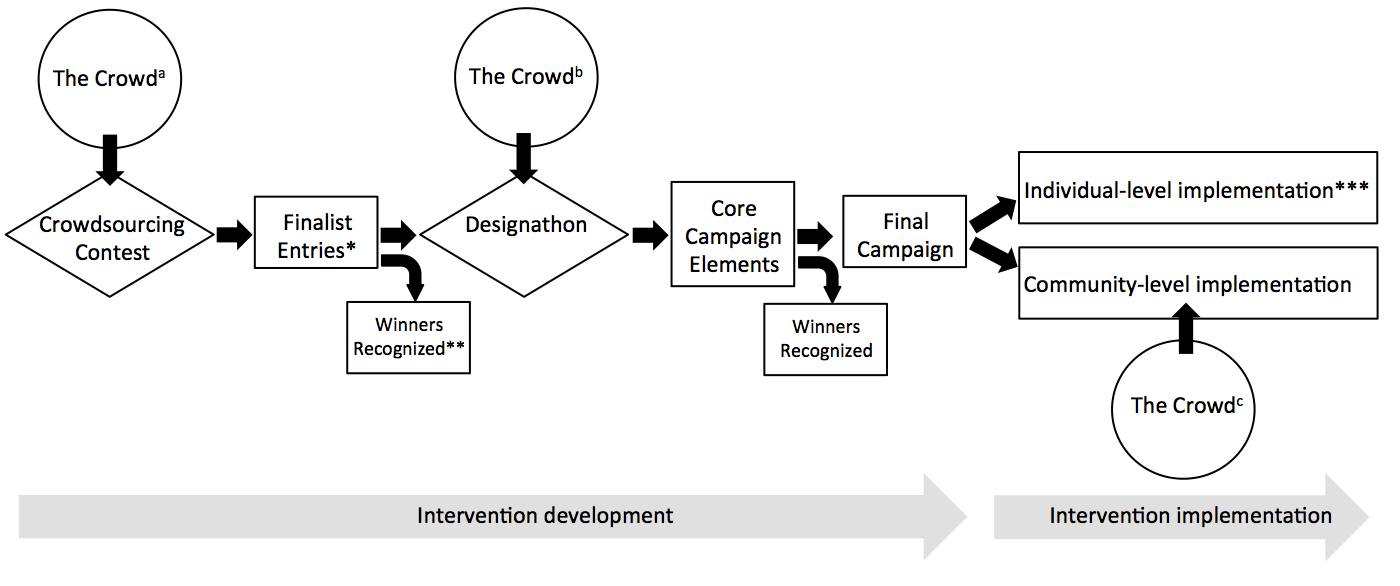
***

*Finalist entries will be selected after an initial screening followed by crowd panel and expert panel judging (Detailed under “Crowdsourcing contest”).

**Crowdsourcing contest winners will be selected based on expert panel scores.

***Individual-level implementation will be divided based on delivery method into two groups: intervention via WeChat message vs. intervention via SMS text message.

a: Crowd involvement in crowdsourcing contest consists of a nationwide, open call for submission of concepts and/or images related to promoting HIV testing among untested individuals.

b: Crowd involvement in designathon consists of multi-sectoral team members working with guidance from professionals to design an intervention plan.

c: Crowd involvement in community-level implementation entails participation in contests and campaign events held by local CDC and CBOs.

*HIV self-testing intervention*

SESH implementation team will send out cohort participants an invitation of receiving free HIV testing kit through a confidential message on social media. Our research team had friended the participants on social media (WeChat) which allows private interaction with them. Individuals who would like to receive this test kit will be directed to an online form that allows them to provide an alias (not their real name), an address, and their cell phone number. These three identifiers will be stored separately in Guangzhou in a locked cabinet. Our research assistant will identify the cohort participants by checking whether the mobile phone number is registered in our baseline survey. Men who provide their address will have a test kit mailed to them. The HIV self-testing package will be sent to participants. The HIV self-test kit is organized in partnership with Guangzhou Tongzhi (GZTZ), a local CBO. The test kits will be offered by SESH. The cover of the mail box will be labeled as “gift”. The mail boxes will be delivered by a courier service. Individuals will be able to implement and interpret the test wherever they want, but will be encouraged to send a photograph of the test result back to our research team through WeChat, which is convenient with a smartphone and has no cost. Individuals who have questions can also contact the study research office in Guangzhou over phone or through social media. Individuals who have a positive test will be able to contact the study research office for information about linkage to care and counseling.

# Statistical Considerations

## Primary Outcomes

The primary outcome of this study will be HIV test uptake over the previous three months. This will be assessed by self-report during follow up survey and triangulated with HIV testing surveillance data from the CDC. A difference of 10% in testing rate (assuming a proportion of HIV testing of 35% during the crowdsourced intervention period and 25% during the conventional intervention period) was chosen based on existing levels of HIV testing and what would be feasible and have public health importance in the Chinese context. The CDC measurement of the primary outcome will be the difference between HIV testing comparing the three months immediately prior to the intervention and the three months of the intervention.

## Secondary Outcomes

A number of secondary outcomes will also be measured. These include syphilis testing, condomless sex, community engagement, testing stigma, and others (Appendix 1). Outcomes will also be stratified based on the level of engagement in developing the intervention, and based on the personal level of engagement during the stage of intervention implementation.

## Sample Size Calculation

We used a binary outcome stepped-wedge randomized controlled trial design for sample size calculation. The required sample size is calculated for the primary outcome. To calculate sample size, we assumed that a crowdsourced intervention will be superior to a conventional method in promoting HIV testing among MSM who never tested for HIV. Assuming a proportion of HIV testing of 35% during the crowdsourced period and 25% during the conventional period, total number of clusters of eight, total number of time period for intervention of four, coefficient of variation of 0.4 (usually between 0.15 and 0.4), 2-sided alpha=0.05, 90% power, and 30% loss to follow up, then the total sample size will be 1040 men (130 for each city). To further improve the power for sub-analysis and secondary outcomes, we will increase the sample size to 1280 men (160 for each city). The calculation was made using the formulas developed by [Michael A. Hussey](http://www.sciencedirect.com.libproxy.lib.unc.edu/science/article/pii/S1551714406000632) et al.[^29^](#_ENREF_29) (<http://faculty.washington.edu/jphughes/pubs.html>).

**Table 2: sample size calculation**

| **P_i_*** | **P_c_^$^** | **Number of clusters** | **Number of time periods** | **Coefficient of variation^@^** | **Alpha** | **Power** | **Sample size/no LTFU** | **Loss to follow up** | **Sample size for each cluster** | **Total sample size (8 clusters)** |
| --- | --- | --- | --- | --- | --- | --- | --- | --- | --- | --- |
| 30 | 20 | 8 | 5 | 0.4 | 0.05 | 0.9 | 80 | 30% | 114 | 912 |
| 30 | 20 | 8 | 5 | 0.15 | 0.05 | 0.9 | 71 | 30% | 101 | 808 |
| 30 | 15 | 8 | 5 | 0.4 | 0.05 | 0.9 | 31 | 30% | 44 | 352 |
| 30 | 15 | 8 | 5 | 0.15 | 0.05 | 0.9 | 26 | 30% | 37 | 296 |
| **35** | **25** | **8** | 5 | **0.4** | **0.05** | **0.9** | **91** | **30%** | 130 | **1040** |
| 35 | 25 | 8 | 5 | 0.15 | 0.05 | 0.9 | 83 | 30% | 119 | 952 |
| 35 | 20 | 8 | 5 | 0.4 | 0.05 | 0.9 | 37 | 30% | 53 | 424 |
| 35 | 20 | 8 | 5 | 0.15 | 0.05 | 0.9 | 31 | 30% | 44 | 352 |
| 40 | 30 | 8 | 5 | 0.4 | 0.05 | 0.9 | 99 | 30% | 141 | 1128 |
| 40 | 30 | 8 | 5 | 0.15 | 0.05 | 0.9 | 93 | 30% | 133 | 1064 |
| 40 | 25 | 8 | 5 | 0.4 | 0.05 | 0.9 | 42 | 30% | 60 | 480 |
| 40 | 25 | 8 | 5 | 0.15 | 0.05 | 0.9 | 36 | 30% | 51 | 408 |
| 45 | 35 | 8 | 5 | 0.4 | 0.05 | 0.9 | 104 | 30% | 149 | 1192 |
| 45 | 35 | 8 | 5 | 0.15 | 0.05 | 0.9 | 99 | 30% | 141 | 1128 |
| 45 | 30 | 8 | 5 | 0.4 | 0.05 | 0.9 | 45 | 30% | 64 | 512 |
| 45 | 30 | 8 | 5 | 0.15 | 0.05 | 0.9 | 40 | 30% | 57 | 456 |

Note: *Pi: probability of HIV testing during intervention period; ^$^Pc: probability of HIV testing during control period; ^@^ usually between 0.15-0.4.

## Analysis Plan

### Primary Outcome Analyses

The primary outcome will be self-reported HIV testing uptake in the past three months. We will examine the hypothesis comparing the superiority of the crowdsourced intervention compared to conventional HIV test uptake campaigns. In our study, since the outcome is binary, generalized linear mixed models (GLMM) and generalized estimating equations (GEE) can be used in our study for the primary outcome analysis. However, since we only have eight clusters, GLMM will be used for primary data analysis in our study; GLMM is preferred in studies with a small number of clusters[^30^](#_ENREF_30). The model will include intervention status and time as fixed effects and site and individuals as random effects. The estimated intervention effects will be reported with 95% CIs and p values. Descriptive analysis will be used to summarize the characteristics and behaviours of the participants at baseline and follow-up surveys.

### Secondary Outcome Analyses

Similar analyses will be conducted for binary secondary outcomes (continues variables will be categorized into binary variable), including frequency of syphilis testing, frequency of HIV testing (among those with previous HIV testing), condomless sex, community engagement, awareness of HIV status, empowerment and others. In addition, since four cities will implement more intensive in-person events to promote engagement (Guangzhou, Shenzhen, Qingdao, and Jinan) during intervention development, sub-analysis will be conducted to evaluate the potential effect of in-person events to promote HIV testing and other secondary outcomes among Chinese MSM. In addition, secondary analysis will investigate an interaction effect between intervention and community engagement (both for engagement during the intervention development stage and engagement during the intervention implementation stage, at personal level).

### Sub-Analyses

Sub-analysis will also be conducted to compare the effect of the intervention in participants with different age (less than 30 versus 30 or older), and to compare two delivery methods for individual-level intervention: WeChat message vs. SMS text message.

# Ethical considerations

## Risk Analysis

### Risks to the patient

Participants in the online cohort may feel embarrassed, anxious, or otherwise distressed by providing information of such a personal nature. Participants may also experience fatigue in response to the proposed evaluations (i.e., from looking at a computer screen).The study consent form will inform participants of confidentiality guidelines. Strict security measures will be put in place to protect privacy and prevent confidentiality breaches (see section 8.1.3)

### Benefits of the study

Participants enrolled in this study are part of a population at high risk of HIV. The intervention of this study may influence participants to get HIV testing, as well as encourage safe sex practices. The most important direct benefit of participating in this study is that participants may obtain an HIV test and seek care after being exposed to the intervention. These men will receive HIV and risk reduction counseling at local VCT sites and care and/or care at ARV clinics. For men who are tested HIV-positive, they will be linked to treatment and care. Potential indirect benefits include psychological and social support, where staff will make appropriate referrals for psychological and social support services in their area. Another potential benefit is that participants may experience the altruistic benefit of participating in a study that contributes to health of MSM, and ultimately strengthening the MSM community in China.

Further, the proposed study will make important contributions to the literature. The field of HIV interventions among young MSM in resource-limited settings is in its infancy. This project will result in the creation of a MSM-targeted community-level intervention that will be fielded and evaluated in the Chinese setting. We will determine whether this type of intervention is acceptable among MSM in China and whether it has effect on increasing VCT uptake and linkage to care among Chinese MSM currently at high-risk for HIV acquisition.

In addition, the proposed intervention can be applied in other settings through other organizations, and can be easily scaled up. By partnering closely with community-based organizations and local public health officials, this project will generate capacity for the MSM community to carry out similar activities and further strengthen the relationship between community-based organizations and the local government.

Understand the effect of this intervention will be important for HIV prevention strategies in China. If the intervention has effect, this will be the first intervention designed to increase HIV testing and linkage among young MSM in China. This study can serve as a starting point for understanding the social and cultural considerations of such an intervention among a marginalized population in a resource-limited setting, and the translation of such an intervention to other communities in Asia and among other key populations.

This is the first study to explore the use of crowdsourcing to improve HIV testing and linkage interventions. The data gathered from this research will help to inform crowdsourcing interventions in other middle-income settings and contexts. Findings from our study will be disseminated to journals focusing on HIV/AIDS, psychology, behavioral science, public health and the social sciences. Papers will be submitted to the American Public Health Association, International AIDS Society, the Asian-Pacific Society on AIDS, and the Society for Epidemiologic Research conferences. Findings will also be disseminated to community-based organizations and local disease prevention agencies in China.

## Confidentiality

For the baseline and follow-up assessments, all data are directly entered into computers as participants complete the surveys. Programs to ensure accuracy, completeness, and internal consistency are automated. Data can be readily downloaded and converted to the format of commercially available statistical software. During collection of the online portion of the study, all data will be transmitted securely using SSL (TLS) 128 bit encryption across the Internet (HTTP). SSL providers users with the assurance of access to a valid, “non-spoofed” site, and prevents data interception or tampering with sensitive information. The SSL certificate that will be used for this project will use 128-bit encryption, the preferred security level of government and financial institutions. 128-bit encryption offers protection that is virtually unbreakable. For example, if a hacker could crack a standard 40-bit SSL session in a day, it is estimated that it would take well beyond a trillion years to accomplish the same thing against a 128-bit SSL session. A dedicated server, which eliminates security issues involved with shared hosting environments where hundreds of websites and users reside on one shared web server as well as ensuring both physical and network security, will be used to house the data. Data will be located in a secured server at UNC Chapel Hill. The server will be configured with redundant hard drive array to ensure reliability. Access to the data will be password protected within the server’s firewall. Survey responses will be kept separately from participants’ email addresses; the two files will be linked with a non-descript, unique, randomly generally identifier. Only the PI and a designated senior staff member will have the password to access to the “key” that links the non-descript identifier to personally identifiable information. IP addresses of participant's computers will not be collected at any time. Cookies will not be used in any way to track participant activity. A quick link will exist on each survey page to provide participants a rapid way to switch to an innocuous website if their privacy is interrupted while completing the survey.

No presentation or publication of the study results will refer to participants individually. Manuscripts published regarding this work will be based on the accumulated database. Exceptions to confidentiality for participants are those required by law and include suspicion of child abuse, elder abuse, and threat of imminent action on suicidal or homicidal ideation. Participants will be informed of these exceptions in the informed consent process.

The potential for deductive disclosure is unlikely. Individual identities and survey responses will not be directly linked to e-mail addresses or telephone numbers, as all identification will be re-coded by using a random number generator.

## Monitoring procedures

In accordance with the NIH Policy for Data and Safety Monitoring (06/10/1998), this research study will have an independent DSMB composed of the following world-recognized HIV experts: Dr. Paul Volberding (DSMB Chair), Dr. Fujie Zhang, Dr. Jinkou Zhao, Dr. George Rutherford, and Dr. William Wong. They will meet and during their initial meeting establish a charter with the following key components: monitoring procedures, frequency of monitoring, content of DSM report, collection and reporting of SAEs and AEs, reporting mechanisms of AEs/SAEs to IRBS and NIH, reporting mechanisms of IRB actions to the NIH, reporting mechanisms for changes or amendments to the protocol or consent form, potential risks and benefits to subjects, management of SAEs or other study risks, conflicts of interest, data acquisition and transmission, data analysis plans, trial stopping rules, and plans for interim analysis. The DSMB will convene in-person at least once per year at CROI or IAS meetings. The frequency of teleconference meetings will be greater during the intervention itself. The study is registered in the Clinical Trials.gov database (NCT02796963).

## Compliance

Dr. Joseph Tucker will lead the development and implementation of the crowdsourcing intervention (and all related Guangdong Province research) and overall study coordination. Dr. Tucker will be responsible for fiscal and research administration. The PIs will communicate weekly, either by phone, email, Skype conference, or in person, to discuss the design, data analysis, subject safety, and all administrative responsibilities. All research results will be shared with PIs, key personnel, and consultants. The Co-PIs will work together to discuss any changes in the direction of the research project.

## Participant withdrawal

There are no specific procedures for withdrawing from the study. Participants are informed that they may withdraw at any time.

## Institutional review board

IRB approval will be obtained from the following institutional ethical review boards prior to study enrolment: Guangdong Provincial Center for Skin Diseases and STI Control, University of North Carolina at Chapel Hill, and University of California San Francisco. The clinical investigator will present study progress reports to the IRB and the sponsor in accordance with government and institutional regulations and in agreement with the policy established with or by the sponsor. The IRB will be informed of any modifications or amendments to the protocol. The clinical investigator will obtain approval in writing from the IRB prior to implementation of any changes that increase subject risk or that alters the objectives of the study or validity of the data collected. The resulting copy of the IRB approval will also be provided to the sponsor.

## Informed consent

Before entering the online survey, participants will be presented with the consent form. Based on standards from previous Internet research studies, the participant will be required to answer 5 questions related to principal elements of informed consent described in the consent document. Participants will indicate their consent by selecting ‘‘I consent and wish to enter the study’’ or ‘‘I do not consent and wish to leave the study.’’ Links to ‘‘tell me more’’ and to ‘‘email the researchers first’’ will also be provided along with more detailed information about the study. The consent document will also explain that completion of the questionnaire will have to occur in one setting. Once participants consent to participate, the study questionnaire will be opened for completion.

For participants under age 18, a waiver of parental permission will be requested. While the age for general consent is 18 years old in China, the age of consent for sex is 14 years old. Excluding this group would decrease our understanding of this vulnerable group. However, obtaining parental consent would effectively "out" participants in the survey to their parents, which could cause psychological harm, condemnation, and family problems.The Chinese IRB which is bound by Chinese laws and regulations has assessed the advantages and disadvantages of requiring parental consent and allowed a waiver of parental consent for 16 and 17 year olds.

Native Chinese speakers will be translating written consent forms to make sure that they are both valid and reliable. Online consent will be obtained using standard procedures developed by the Guangdong Provincial STI Control Center. The consent form will contain all elements of a written consent form required by the IRB of the Guangdong Provincial STI Control Center, including the purpose of the study, the investigators, all procedures, the risks and benefits, and contacts for further information or to lodge complaints.

Participants will not be required to sign a written consent form. The proposed research questions present no more than minimal risk of harm to subjects and involve no procedures for which written consent is normally required.

# Appendix 1. Secondary outcomes measured as part of this RCT.

| **Secondary Outcome** | **Definition** |
| --- | --- |
| *Incremental cost (overall)* | Incremental cost, defined as the cost associated with respective interventions (development, start-up, implementation, condom use, intervention per individual who reported no sex or sex with a condom during the follow-up period.) |
| *Incremental cost for HIV self-testing* | Incremental cost for HIV self-testing, defined as the cost associated with HIV self-testing (development, start-up, implementation, condom use, intervention per individual who reported no sex or sex with a condom during the follow-up period.) |
| *Condom use* | Frequency of men, defined as the number of men who reported increased condom use (in anal, vaginal, and/or oral sex with either male and female sex partners) comparing pre-intervention and post-intervention values |
| *HIV testing social norms* ^1^ | Frequency of men, defined as number of men who report higher levels of social norms when comparing their pre-intervention and post-intervention HIV testing norms |
| *HIV testing self-efficacy* ^2^ | Frequency of men, defined as number of men who had an increase in HIV testing self-efficacy when comparing their pre-intervention and post-intervention self-efficacy |
| *Community engagement/ MSM community affiliation* | Frequency of men, defined as an increase in closer affiliation with the MSM community (i.e., tongzhi circle, gay online networks or groups) when comparing their pre-intervention and post-intervention engagement |
| *Campaign engagement* | Frequency of men, defined as number of men who had an increase in taking part in the HIV testing campaign when comparing their pre-intervention and post-intervention engagement |
| *HIV self-testing (self-report)* | Frequency of men, defined as the number of men who reported being self-tested for HIV during the previous three months |
| *HIV self-testing (confirmed)* | Frequency of men, defined as the number of men who received HIV self-test kits during the previous three months |
| *Anticipated HIV stigma* ^3^ | Frequency of men, defined as number of men who report anticipated HIV stigma when comparing their pre-intervention and post-intervention |
| *Syphilis testing* | Frequency of men, defined as the number of men who reported being tested for syphilis (excluding HIV) during the previous three months |
| *Weibo engagement* | Frequency of men, defined as the number of men who reported using Weibo in the past three months to give or receive information about HIV testing comparing their pre-intervention and post-intervention engagement (Except the intervention delivered by SESH) |
| *Wechat engagement* | Frequency of men, defined as the number of men who reported using Wechat in the past three months to give or receive information about HIV testing comparing their pre-intervention and post-intervention engagement (Except the intervention delivered by SESH) |
| QQ engagement | Frequency of men, defined as the number of men who reported using QQ in the past three months to give or receive information about HIV testing comparing their pre-intervention and post-intervention engagement (Except the intervention delivered by SESH) |
| Mobile app engagement | Frequency of men, defined as the number of men who reported using mobile apps in the past three months to give or receive information about HIV testing comparing their pre-intervention and post-intervention engagement |

^1^ HIV testing social norms will be measured using six survey items that are each on a five point Likert scale. Increased HIV testing social norms will be defined as having an increase from baseline in any two of these six survey items and dichotomized accordingly (social norm change will be categorized into three groups: increased, stable and decreased social norm). The HIV testing social norm outcome will be assessed in the entire group as well as the subgroup of men who were referred by their friends. This is adapted from Pettifor, A., MacPhail, C., Suchindran, S, & Delany-Moretlwe, S. (2015). Factors associated with HIV testing among public sector clinic attendees in Johanesburg, South Africa. AIDS and Behavior, 14, 913-921.

^2^ Self-efficacy will be measured using six survey items that are each on a five point Likert scale. Increased self-efficacy will be defined as having an increase from baseline in any two of these seven survey items and dichotomized accordingly (self-efficacy change will be categorized into three groups: increased, stable and decreased self-efficacy). This is adapted from Gu, J., Lau, J. T. F., & Tsui, H. (2011). Psychological factors in association with uptake of voluntary counselling and testing for HIV among men who have sex with men in Hong Kong. Public Health, 125, 275-282.

^3^ The Anticipated HIV Stigma will be assessed using 7 survey items that are each on a four point Likert scale. Reduced anticipated HIV stigma will be defined as having a decrease from baseline in the mean score (Continuous variable). This is adapted from Golub, S. A. & Gamarel, K. E. (2013). The impact of anticipated HIV stigma on delays in HIV testing behaviors: Findings from a community-based sample of men who have sex with men and transgender women in New York City. AIDS Patient Care and STDs, 27(11), 621-627.

# References

1. He N, Detels R. The HIV epidemic in China: history, response, and challenge. Cell Res 2005;15:825-32.

2. 2015 China AIDS Response Progress Report, National Health and Family Planning Commission of the People’s Republic of China. 2015.

3. Tang W, Han L, Best J, et al. Crowdsourcing HIV Testing: A Pragmatic, Non-Inferiority Randomized Controlled Trial in China. Clinical Infectious Diseases 2016;62:1436-42.

4. UNAIDS. The Gap Report2014.

5. HIV/AIDS: Men who have sex with men. 2015. at <http://www.who.int/hiv/topics/msm/en/>.)

6. Beyrer C, Baral SD, Griensven F, et al. Global epidemiology of HIV infection in men who have sex with men. Lancet 2012;380.

7. Beyrer C, Andrea L. Wirtz, Damian Walker, Bejamin Johns, Frangiscos Sifakis, and Stefan D. Baral. The Global HIV Epidemics among Men who have Sex with Men. Washington DC: World Bank; 2011.

8. Tucker JD, Wong FY, Nehl EJ, Zhang F. HIV testing and care systems focused on sexually transmitted HIV in China. Sex Transm Infect 2012;88:116-9.

9. Li X, Lu H, Ma X, et al. HIV/AIDS-related stigmatizing and discriminatory attitudes and recent HIV testing among men who have sex with men in Beijing. AIDS Behav 2012;16:499-507.

10. Neilands TB, Steward WT, Choi KH. Assessment of stigma towards homosexuality in China: a study of men who have sex with men. Archives of sexual behavior 2008;37:838-44.

11. Wei C, Cheung DH, Yan H, Li J, Shi L-e, Raymond HF. The Impact of Homophobia and HIV Stigma on HIV Testing Uptake among Chinese Men Who Have Sex with Men: A Mediation Analysis. Journal of acquired immune deficiency syndromes (1999) 2016;71:87-93.

12. Zou H, Hu N, Xin Q, Beck J. HIV testing among men who have sex with men in China: a systematic review and meta-analysis. AIDS Behav 2012;16.

13. Chow EP, Wilson DP, Zhang L. The rate of HIV testing is increasing among men who have sex with men in China. HIV medicine 2012;13:255-63.

14. Zhang L, Gray RT, Wilson DP. Modelling the epidemiological impact of scaling up HIV testing and antiretroviral treatment in China. Sex Health 2012;9:261-71.

15. Bull SS, Levine D, Black SR, Schmiege S, Santelli J. Social Media–Delivered Sexual Health Intervention: A Cluster Randomized Controlled Trial. American journal of preventive medicine 2012;43:467-74.

16. Wei C, Herrick A, Raymond HF, Anglemyer A, Gerbase A, Noar SM. Social marketing interventions to increase HIV/STI testing uptake among men who have sex with men and male-to-female transgender women. The Cochrane database of systematic reviews 2011:Cd009337.

17. Swan M. Crowdsourced health research studies: an important emerging complement to clinical trials in the public health research ecosystem. Journal of medical Internet research 2012;14:e46.

18. Naslund JA, Aschbrenner KA, Marsch LA, McHugo GJ, Bartels SJ. Crowdsourcing for conducting randomized trials of internet delivered interventions in people with serious mental illness: A systematic review. Contemporary clinical trials 2015;44:77-88.

19. Surowiecki J. The Wisdom of the Crowds. New York: Anchor; 2004.

20. Parvanta C, Roth Y, Keller H. Crowdsourcing 101: a few basics to make you the leader of the pack. Health promotion practice 2013;14:163-7.

21. Ranard BL, Ha YP, Meisel ZF, et al. Crowdsourcing--harnessing the masses to advance health and medicine, a systematic review. Journal of general internal medicine 2014;29:187-203.

22. Tang W, Han L, Best J, et al. Crowdsourcing HIV Test Promotion Videos: A Noninferiority Randomized Controlled Trial in China. Clin Infect Dis 2016;62.

23. Tang W, Mao J, Liu C, et al. Reimagining Health Communication: A Non-Inferiority Randomized Controlled Trial of Crowdsourcing in China. Durban: International AIDS Society; 2016.

24. Zhang W, Schaffer D. Qualitative Evaluation of Community Engagement as part of an Innovation Contest. Guangzhou2015.

25. Hemming K, Haines TP, Chilton PJ, Girling AJ, Lilford RJ. The stepped wedge cluster randomised trial: rationale, design, analysis, and reporting. BMJ : British Medical Journal 2015;350.

26. Tang W, Mao J, Liu C, et al. Reimagining Health Communication: A Non-Inferiority Randomized Controlled Trial of Crowdsourcing in China. International AIDS Society. Durban, South Africa2016.

27. Walker A, Ko N. Bringing Medicine to the Digital Age via Hackathons and Beyond. J Med Syst 2016;40:98.

28. DePasse JW, Carroll R, Ippolito A, et al. Less noise, more hacking: how to deploy principles from MIT's hacking medicine to accelerate health care. Int J Technol Assess Health Care 2014;30:260-4.

29. Hussey MA, Hughes JP. Design and analysis of stepped wedge cluster randomized trials. Contemporary clinical trials 2007;28:182-91.

30. Hemming K, Lilford R, Girling AJ. Stepped‐wedge cluster randomised controlled trials: a generic framework including parallel and multiple‐level designs. Statistics in medicine 2015;34:181-96.
