# Supplementary material for: Crowdsourcing to expand HIV testing among men who have sex with men in China: A closed cohort stepped wedge cluster randomized controlled trial
Source: PLoS Med. 2018 Aug 28;15(8):e1002645. doi: 10.1371/journal.pmed.1002645 (PMC6112627; doi:10.1371/journal.pmed.1002645)
Supplement: S2 Text — (DOCX) [file pmed.1002645.s002.docx]

# S2 Text. Crowdsourcing Intervention Implementation

***Summary***

The crowdsourcing intervention included elements from a nationwide open contest to solicit images that promote HIV testing, a regional strategy designathon to develop HIV testing strategies, and local participatory contests soliciting HIV testing messages.

***Intervention implementation***

The first part of the intervention comprised images and messages, as well as HIV testing site information, disseminated through personal WeChat messages. During the three-month intervention period, we sent private WeChat messages containing one of the six images, along with local testing site information (location, hours, availability of free testing) to participants once every two weeks. Participants were encouraged to HIV test at local facilities.

| **Images** | **Text in the image** | **Message sent along with the image** |
| --- | --- | --- |
| 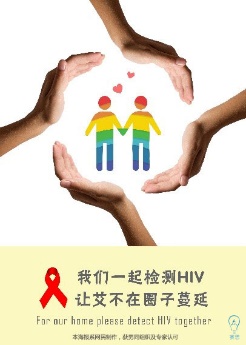 | Let’s test for HIV together.  Stop HIV from spreading in our community. | One community, one dream. We dream that this community will no longer be threatened by HIV. Protect yourself and protect others. Let’s test for HIV together and stop AIDS from spreading in our community! |
| 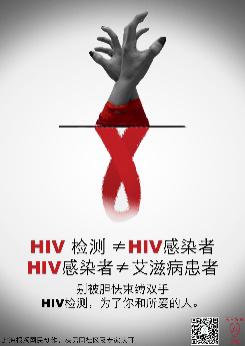 | HIV infected ≠AIDS patients.  Don’t be bound by fear. Get an HIV test for your sake and the sake of your loved one. | HIV testing is not equal to HIV infection. HIV infection is not equal having AIDS. Free your hands. For the sake of you and your loved ones, let’s get tested! |
| 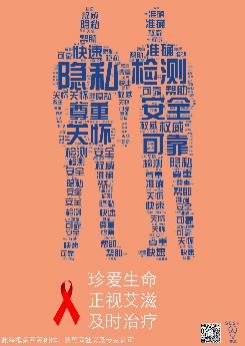 | Keywords in the word cloud: privacy, testing, respect, safety, care, reliable, rapid, accuracy.  Cherish your life; stand up to HIV/AIDS; get timely treatment | The best love originates from cherishing each other. HIV testing is deidentified, safe, accurate, and reliable. Early detection, early prevention and early treatment will lead to long-lasting romance and long-lasting life. |
| 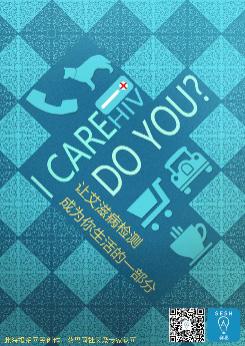 | Let HIV testing become a part of your life. | Like having a cup of tea in the morning, going on a shopping spree, or getting word from an old friend, HIV testing can be a part of your daily life. |
| 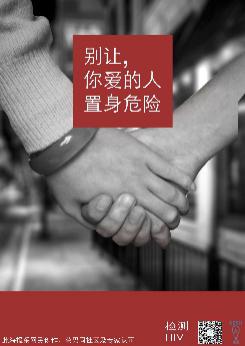 | Don’t put your loved ones in danger. | It might look cool to live without regard for others, but life isn’t complete without caring for your loved ones. Don't put your loved ones in danger: get an HIV test! |
| 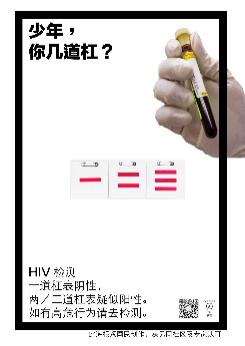 | Son, what’s your rank?  HIV test: one line means negative; two or three lines means suspected positive.  Please go and get HIV tested. | Hey kid, what was your rank in the Young Pioneers of China? In the HIV testing result, one band means negative, two or three bands means suspected positive. Please be proactive about getting tested! |

The second part of the intervention was an HIV self-testing platform. During the intervention period, we sent one WeChat message to the participants offering a free HIV oral self-testing kit. This WeChat message contained a link to an online form, open for one week, through which participants could provide their mailing addresses and apply for the testing kits. Afterwards, we mailed testing kits to all participants who had provided their mailing addresses.

The third part of the intervention consisted of local HIV testing story contests, held in each of the eight participating cities, to engage the local community, especially MSM, on the topic of HIV testing. The SESH team developed an implementation guide for contest organizers. Local CBOs organized these contests with assistance from the local CDC and the SESH team. CBOs also formed a steering committee. This committee designed promotional materials, disseminated the contest to the local MSM population both online and offline, judged the submissions, celebrated the finalists, and shared winning results with the community. CBOs sent at least eight online push notifications to the local community using their social media platforms (websites, Weibo, Blued, WeChat public account, or QQ). CBOs were given the flexibility to design and conduct various offline promoting events -- displaying posters and distributing flyers at VCT clinics and common MSM gathering places, organizing entertainment activities, for example, a live show containing promoting messages <https://v.qq.com/x/page/f0396q6qluq.html>). Submissions were evaluated by invited crowd judges and experts in public health, health communication and sociology. High-quality submissions were shared with the local MSM community on social media platforms.

***Sample social media call for local message submissions***

1. Story and message contest criteria
   1. Story submission
      1. Theme: Related to HIV testing
      2. The story can be yours, your friend’s, or a story you have heard; The story can be a long narrative or as short as three lines. The story can be poignant, repentant, or can blow your mind; In short, there are no limits on the story style or type. If you need a platform and an opportunity to share stories related to AIDS (especially HIV testing or self-testing), this is your stage!
   2. Message
      1. Theme: Related to HIV testing
      2. Messages could be meaningful, humorous, and creative. Messages can be either fiction or nonfiction, hyperbolic or realistic. In short, whether you are stern or humorous, you are welcome to share your AIDS-related (especially HIV testing or self-testing) message.
   3. Submission period
   4. How to submit?
      1. Denote whether the submission is a story or a message, attach the submission with a nickname and contact information of choice (mobile number, WeChat, Weibo, QQ… can also submit with a nickname)
      2. Send to our mailbox [give an email address] or directly respond on our social media platforms.
   5. Selection criterions
      1. The judges invited by the organizing committee will select 10-20 semi-finalists, and publish the semi-finalist and finalists on various platforms.
   6. Awards
      1. Story award

First prize, 800 yuan or equivalent gift value

Second prize, 600 yuan or equivalent gift value

Third prize, 500 yuan or equivalent gift value

- - 1. Message award

First prize, 800 yuan or equivalent gift value

Second prize, 600 yuan or equivalent gift value

Third prize, 500 yuan or equivalent gift value

- 1. Contacts: [Local email and telephone contacts]
  2. Notice: Work must be original, and plagiarized entries will result in disqualification and other consequences.
