# Supplementary material for: Crowdsourcing to expand HIV testing among men who have sex with men in China: A closed cohort stepped wedge cluster randomized controlled trial
Source: PLoS Med. 2018 Aug 28;15(8):e1002645. doi: 10.1371/journal.pmed.1002645 (PMC6112627; doi:10.1371/journal.pmed.1002645)
Supplement: S3 Text — (DOCX) [file pmed.1002645.s003.docx]

# S3 Text. Three stories from Community Story Contest

| 故事1：《第一次去深圳258彩虹工作组检测经历》  记得是2009年，那时刚入圈不久，经常浏览深圳当地通知交友网站，看到了深圳同志公益组织258彩虹工作组有免费检测HIV的项目。那时住布吉，离258彩虹工作组所在地东门很近，当时犹豫了很久才决定去检测。当然检测是一方面，另一方面还想看看这样一个同志公益组织是什么样子。到了东门胡润大厦很好找，坐电梯到了22楼，电梯门刚打开就看到两个男孩子貌似在吻别，凭直觉就知道是对同志情侣。第一次来这样的地方心里真还有点噗通噗通忐忑不安。找到2213房间，房间布置的简单温馨，里面坐着两三个男孩子，也见到了之前网上看到过的彩虹工作组负责人卡其，后来了解到那几个男孩子都是志愿者。说明来意，其中一个男孩子拿来一份调差问卷，填了之后就去里面的小房间，市疾控中心医生在那值班抽血检测，检测完全是保密的，每个人都有一个编码，三天后可以在深圳同志交友网站上查询。抽完血，稍微坐了会，和其中一个志愿者聊了会，258彩虹工作组有了进一步了解，他们周末经常去同志聚集的公园，酒吧宣传防艾知识，免费发放防艾资料，安全套。组织志愿者有部分人也是感染者。但是他们看起来都很阳光，生活积极向上。聊天期间，陆续又有人来检测。坐了一会准备回去，还发了几盒套套和宣传资料。记得那时还发了50元路费。回去等结果的三天真的是无比煎熬的三天，当看到检测结果阴性，一颗悬着的心终于放了下来。虽然几年过去了，这段经历记忆还是挺深刻的。几年过去了，身边也有感染的，有的甚至不知道被谁感染的，他们也经历了痛苦的挣扎，失望的哭泣，值得一提的是，经过一番痛苦的挣扎，由绝望到接受，现在他们都生活的好好的，甚至比以前生活的更好，因为他们更努力，更懂得珍惜生活，懂得了健康的重要。身为同志，这些年我深有感触的是像深圳这些一线大城市，防艾宣传工作做的很好，也有一些同志公益组织做工作，起码这样的城市的同志防艾意识还是比较强的，而一些三四线城市则不同，消息闭塞，防艾思想意识不强，甚至根本没这个意识，小城市里生活的同志都很隐蔽，其实约炮，滥情情况更严重，更可怕的是很多没有安全保护意识，即使感染了自己都不知道，很多都是生病去医院抽血化验才知道或者献血才查出来。更甚者有些自暴自弃的感染者明知道自己是感染者，依然和别人危险性行为，甚至和女的结婚。所以，三四线城市防艾的工作应该也要重视，多些像深圳258彩虹工作组这样的同志公益组织，多深入到同志人群中宣传防艾知识和安全性行为，以及对感染者心理干预，让生活在小城市的同志人群防艾意识提高起来！ | Story 1: "The first testing experience at the Shenzhen 258 Rainbow Workgroup "  I remember it was 2009, when I just joined the circle. I would often browse Shenzhen local social network websites, and I saw the Shenzhen non-governmental organization, 258 Rainbow Workgroup, offers free testing for HIV. At that time, I lived in Buji, very close to the east gate of the 258 Rainbow Workgroup, but I hesitated for a long time before deciding to go for a test. Of course, the act of testing is one aspect. On the other hand, I want to also see what this kind of LGBTQ organization looks like. The East Gate Hurun building was very easy to find, I took the elevator to the 22nd floor, as the elevator door opened I saw two men seem to be kissing goodbye, by intuition I believe it is a gay couple. The first time I came to such a place, I felt a little uneasy. I then found room 2213, the room is simple and warm, there are two or three men in it, and Kaqi, the head of the 258 Rainbow Workgroup, who I recognized from their website, and later I learned that the men were all volunteers. Next, one of the boys gave a questionnaire to me, after filling the survey, I was invited into a small room. Inside is the CDC doctor on duty who test the blood, the test is completely confidential, each person has a code, and three days after we can see the results on the Shenzhen LGBTQ social network website. After the blood test, I sat in the to talk with one of the volunteers to talk about the organization. I learned that during the weekend, the 258 Rainbow Workgroup often go to the parks and bars where LGBTQ individuals frequent to spread AIDS knowledge, and distribute free condoms. Some of the volunteers in the organization were also HIV-positive. But they look very bright, energetic and live positively. During our chat, one after another came in for testing. When I was ready to leave, they gave me a few boxes of condoms and health information materials. I remember also receiving 50 yuan as a transportation stipend. The three days of waiting for the test results were the three days of unparalleled torment. When the test result was negative, my unsettling heart finally came down. Although several years have passed, this experience is still quite impactful. A few years later, there are still individuals infected, some even do not know who infected them, they have experienced painful struggles, disappointing weeps. It is worth mentioning though, after a painful struggle, from despair to acceptance, and they now live better, or even better than before, because they work harder. I am more appreciative of life and I know the importance of health. As LGBTQ individual, I have a deep feeling for the AIDS work in large city such as Shenzhen. However, in third and fourth tier cities, this is different. There are lack of knowledge and promotion for AIDS. The LGBTQ individuals are hidden, some not even knowing their status until they go for a blood test for other reasons at the hospital or they try to donate blood. Even more, some horrendous HIV-positive individuals know the status, but still has risky sexual behaviors with others, and even marry women. Therefore, third or fourth tier cities should pay attention to AIDS prevention work, like the Shenzhen 258 Rainbow Workgroup. They need more public welfare organizations to publicize the knowledge and safety of AIDS, and the psychological intervention for the infected, so that the people living in small cities to improve their understanding of AIDS! |
| --- | --- |
| 故事2：《直面艾滋，不所畏惧》  我叫小尚，今年18岁，是“艾滋病毒”携带者。  临近开学，我去当地的检测中心去进行检测。不幸的是我被检测出“艾滋病”。听到检测结果的的那一刻，犹如晴天霹雳，瞬间感觉全身麻木了，头皮都在发麻，全身都在一个劲的哆嗦，感觉全世界都塌了。心里一个劲的后悔，“我才18岁啊，我的大好青春，我的前程都会被毁于一旦。我该怎么去面对我的家人，朋友和同学，他们该如何看我？” 心里百般无奈。眼泪在眼眶中打转，眼泪里夹杂着，后悔，难过，害怕，恐惧.......等等。检测员安慰着我，让我振作，不要去害怕，让我勇敢起来去积极的面对， 并且好好的配合治疗。  这件事情发生在我生日的时候。这一天，我一如既往的打开“Blued”。一条消息弹出在我的手机屏幕上，内容是：生日快乐，“我的宝贝！”当时我就好好奇，“这个人为啥就知道我的生日呢？而且还要请我吃饭呢？”在我再三追问下，他说出了原委。说来也是缘分，我认识这个人，他是我同学的哥哥，因为我经常去他们家学习，所以都认识了，因此他是从他弟弟口中了解我的情况。当时他已经是病毒的携带者，只是他的家里人都不知道。聊了许久，才答应跟他出去陪我过生日。他比较强势，还有点霸道，挺阳光开朗的，本身就是对他有点好感，所以在他再三推托下我就接受了他的邀请了。  去了他预订好的餐厅，他说：“今天我生日，让我稍微的喝点酒。”可是我是不会喝酒的，我是属于那种只要一沾酒就最的呢种人，而且喝醉后我就会短暂的失忆，不记得喝酒前的任何事情。在他的再三推托下，我只好硬着头皮喝，越喝越多知道喝不下去直接趴在桌子上。  可想而知，在我最后发生了什么，才导致自己变成了携带者。终于知道，不能随随便便接受别人的邀请，要好好的保护自己。  面对检测结果，我也认命了，可能这就是命。尽管自己是多么的后悔，多么的无奈，可是事情已经发生能怎么样！只是我不知道我该如何是好？不知该如何去面对同学的目光。不知该如何向父母交代自己是“艾滋病毒”的携带者。不知该如何面向自己身边的朋友。我感觉我的世界都开始变得灰暗起来，就连我的性格都发生了改变，变得怪癖，暴躁无比。父母经常对我说：“儿子，你最近这么了，为什么会变成这个样子？”慢慢的我也想开始放弃自己。  当我认识他们的时候，他们都是“HIV”的携带者，他们照样活得很好，阳光开朗。他们没有害怕，没有恐惧，他们积极接受治疗，跟正常人没有什么两样。他们每个人都给我讲述他们的经历，目的就是要让我重新振作起来，不能放弃自己。他们的故事给我莫大的鼓舞，也给我直面面对“HIV”病毒的勇气。是他们给我希望，给了我让我活下去的勇气。正因为他们给了我勇气，我也鼓起了勇气欣然面对这一切，并把这一切告知了父母。刚开始父母各种的不理解，这种的讽刺，都让我心灰意冷。可是我还想起我还有这样的一群朋友，我就有了动力，在我的努力下，父母终于接受了我是“艾滋病毒”携带者这个事实。现在父母经常在开导和鼓励我。让我有了活下去的坚定信念。  父母在一夜之间白了头，为了我操碎了心。所以我要坚强的活下去，不能让他们在为我担心了。  爸妈，我会勇敢的活下去，不会让你们为我担心了。  我们要勇敢，乐观开朗的活下去，不能自我放弃。  我们要加油。  我们要直面“HIV”，不所畏惧！ | Story 2: <To face AIDS, not to fear>  My name is Xiao Shang. I am 18 years old. I am a “carrier” of HIV.  Near the beginning of school, I went to the local testing center for HIV testing. Unfortunately, I was tested positive for HIV. Hearing the results at the moment, like a bolt from the blue, I felt numb, even the scalp is numb – the whole body starts to shiver intensely. I felt the whole world collapsed. Regret in my heart, "I am only 18 years old, my great youth, and my future will be destroyed." How should I face my family, friends and classmates? How would they see me? " My heart felt helpless. Tears rolled in the eyes, tears mixed, regret, sadness, fear, fear, and so on. The testing staff comforted me, told me not be afraid, let me be brave to face the result positively, and cooperate well with the treatment.  This happened on my birthday. That day, I open the "Blued" as usual. A piece of news popped up on my mobile phone screen. The content is: happy birthday, "my baby!" I was so curious at that time, "why does this person know my birthday?" And asked to take me out to eat? " When I asked again and again, he told the truth. It is also fate. I know this person, he is my classmate's brother, because I often go to their home to learn, so they all know me. At that time, he was already a carrier of the virus, but his family did not know. After a long chat, I agreed to go out with him to celebrate my birthday. He is more dominate, and he is a little overbearing but sunny. I was somewhat interested in him, so I accepted his repeated invitation.  He went to the restaurant he ordered, and he said, "let you have a little drink today for your birthday." But I do not drink, I am the kind of person who has very low drinking tolerance, and I will be short of memory after drunk, I will not remember anything before drinking. In his repeated push, I drink hard and continue drinking more and drink more, and fell directly on the table.  You can imagine what happened to me next that made me a carrier. Thus, one should not accept others' invitation casually and protect oneself.  In the face of the test results, I also accepted my fate. Maybe this is fate. No matter how regretful and helpless I am, what has happened has happened. Just I don't know what I'm going to do? I do not know how to face the eyes of the other students. I don't know how to tell my parents that I am a carrier of HIV. I don't know how to face my friends. I feel that my world is beginning to turn grey, and even my personality has changed and become quirky and irascible. My parents often say to me, "son, why are you like this recently?" Slowly I also wanted to give up myself.  When I met them, they were all carriers of "HIV". They still lived very well, and were very sunny. They are not afraid, no fear, they are actively receiving treatment, and are no different to normal people. Each of them told me their experiences; the purpose is to motivate me to not give up on myself. Their stories gave me great encouragement and gave me the courage to face the HIV virus. They gave me hope and gave me courage to live. It was because they gave me courage that I summoned up courage to face all this and told my parents. At first my parents did not understand, and their sarcasm made me frustrated. But I knew that I still have a group of friends, I have a motivation, and in my efforts, my parents finally accepted the fact that I was a HIV carrier. My parents are often enlightening and encouraging me now. Let me have the motivation to live.  My parents’ hair turned white overnight, because they worry about me. Therefore, I must be strong and continue to live, and not to let them worry.  Mom and dad, I will be courageous and continue to live, I will not allow myself to go.  We need to cheer up.  We need to directly face “HIV” with no fear! |
| 故事3：阳性爱情  树叶黄了，秋天到了，给自己一个微笑，继续往前走。背起行囊，趁月亮哥哥还在，我松松衣带，默默的对自己说：我走了，我的宋池哥…                     人生的轨道上，有些人跑，有些人走，还有些人走不了只有爬。我就是第三个，而且还是一点点向前爬。可是自从遇见我的宋池哥，我觉得这些年所有的酸甜苦辣都是值得的。  又整理了下衣服，我的手下意识的碰到了那张该死的纸…那是一张医院的艾滋病检测报告，阳性。我死死的盯着那两个字，仿佛要把它在毁灭在我眼里。不行，我不能死，我才23岁，我还有很长的路没走完，我还没有报答我那可怜的双亲，我还有很多很多的事情没有完成！我不敢相信这是真的，这一切都是做梦，快点醒过来…于是我像疯了一样跑了起来。  可是现实就是现实，待我跑累了，我还是我，没有从梦中醒来。三年前，我和我的宋池哥相见于网络上一个名叫BL的同志交友软件。某天的一个晚上，我收到了他的第一声问候，而我随便看了一眼就没理他。第二天晚上同一时间我收到了他的第二句问候，我就随便发个问候语回复了一下，到第三天的时候我们已经聊的水深火热了。  一个月后某天，我见到了他。他站在阳光下，脸上没有一丝阴影，傻傻的憨厚的笑着，仿佛千年前就已经认识的一个朋友，又若是在哪里见过的一个熟友。我就那样站在他面前，把自己笑的一脸的孤寂…  两年后的某天， “哥，我们去做检测吧！对你对我都有好处！”  “不去！我身体健康着呢！”  “不是，这是艾滋病检测，与健康不健康没多少关系！就算是健康，检测一次也没有坏处吧"  最后，我的宋池哥还是被我软磨硬泡的拉到了医院。可是谁知道，他是健康的，我却是阳性……过往种种，仿佛就在眼前，又仿佛很遥远，而现在一切都是空谈…我不知道是谁传染给我的，而我也懒得去追究，我的心已经死去，我只想找个安静的地方，与孤独为伍，让寂寞陪伴。可是我终究放不下你，我的宋池哥。我又怎么放的下你呢？你有胃病，要注意吃饭，不能饿着，你每天都熬夜，没我在一定要早睡早起，你不爱穿太长的袜子，你喜欢吃辣的…  电话想了，那是一个多么熟悉的号码，可我不敢接，我怕接了以后就没勇气面对。十点了，城市的喧嚣已经在路灯下渐渐沉睡，我翻了下手机，一共二十八个未接电话，18条未读信息，全部是我的宋池哥的…前面不远处是一座公园，是我和我的宋池哥经常去的地方，我拖着行李箱在一处寂静的地方做了下来，路还是那条路，可是人已经不是那个人。昏暗的路灯下，我无意中又打开了他的信息，一看可把我乐坏了：“弟弟，快回来吧，医院刚说他们只是初步筛查，你的确证检测结果是阴性，你没有被感染…”我简直不敢相信自己的眼睛，这一切像都是一场梦，那么真切，又那么模糊……  如今六年已经过去了 ，我和我的宋池哥依然在一起。我们约定每半年做一次检测，这是对我对他都有必要的事，而他也养成了去做检测的习惯。迄今为止，我们都很健康。我很高兴那次的检测经历，虽然过程很痛苦，但是我学会了感恩，懂得了珍惜。艾滋检测是对他对自己都有好处的事情，同时这也是一种对生活负责的态度。         2016年，我们去了很多地方，西藏，海南…都留下了我们爱的足迹。2017年，我和我的宋池哥要去一个地方，那里草长莺飞，碧海潮生. | Story 3: Positive love  The leaves are yellow and autumn is here. Give yourself a smile and continue to move on. Pick up the bag, while the moon brother is still there. I loosened my belt and silently said to myself, "I left, my brother Song Chi..." On the track of life, some people run, some people walk, others still cannot walk but climb. I am the third, and I climb a little bit. But since I met my brother Song Chi, I think all the joys and sorrows of these years are worth it.  Finished organizing the clothes, my subconscious ran into the goddamn piece of paper. That is a report showing “HIV positive” from the hospital. I stared at the two words, as if to destroy it in my eyes. No, I can't die. I'm 23 years old. I still have a long way to go. I haven't paid my poor parents yet. I still have a lot of things that I haven't finished! I can't believe it's true. It's all a dream. Wake up quickly. So I ran like crazy.  But reality is reality. When I'm tired of running, I still don't wake up from my dream. Three years ago, I met my brother Song Chi on the internet, a friending network called BL. One night, I received his first greeting, and I took a casual look and ignored him. I received second greetings from him at the same time on the second night, and I gave a reply to the greeting, and by the third day we had a deep conversation.  Some day in the following month, I saw him. He stood in the sun, with no shadow on his face, a silly smile, as if a friend who I have known from thousand years ago. So I stood in front of him and smiled with my lonely face.  Some day after two years, "brother, let's go get tested. It's good for you for me! "  "No!" I'm in good health! "  "No, this is HIV testing. It has nothing to do with unhealthy health. Even if you’re healthy, there's no harm to test once. "  In the end, I dragged my brother Song Chi to the hospital. But who knows, he is healthy, but I am positive... All the past, as if in front of us, seemed very distant, and now everything is empty talk. I did not know who has transmitted to me, and I also was too lazy to investigate. My heart had died; I just wanted to find a quiet place, with loneliness, let loneliness be my companion. But I can't bring you down, my brother Song Chi. How do I bring you down again? You have a stomach disease, should pay attention to eating, cannot be hungry, you stay up every night, I must be early to sleep early, you do not like to wear too long socks, you like to eat spicy...  The phone rang, and it was a familiar number, but I didn't dare to answer it. I was afraid I would not have the courage to face it. It was ten o’clock, the hustle and bustle of the city has been sleeping under the light of the street, I turned on the phone, twenty-eight unanswered phones, 18 unread information, all from my brother Song Chi. Not far from the front is a park, a place where I and my brother Song Chi often go. I dragged the suitcase down in a quiet place, this road or that road, but the man had become a different man. Under the darkened streetlight, I unintentionally opened his message again. "My brother, come back soon, the hospital just said they were just preliminary screening, your confirmatory test results are negative, you have not been infected..." I can't believe my eyes. It's all like a dream, so real and so vague...  Now six years have passed, and me and my brother Song Chi are still together. We agreed to do test every six months, which is necessary for me, and he also developed the habit of doing tests. So far, we are all very healthy. I am grateful for the testing; although the process is very painful, but I learned to be grateful, know how to cherish. HIV testing is good for one, and it is also a responsible attitude towards life.  In 2016, we went to many places, Tibet, Hainan... All the footprints of our love have been left at these places. In 2017, my brother and I will go to a place where the grass grows tall and the blue sea waves. |
