# Supplementary material for: Crowdsourcing to expand HIV testing among men who have sex with men in China: A closed cohort stepped wedge cluster randomized controlled trial
Source: PLoS Med. 2018 Aug 28;15(8):e1002645. doi: 10.1371/journal.pmed.1002645 (PMC6112627; doi:10.1371/journal.pmed.1002645)
Supplement: S4 Text — (DOCX) [file pmed.1002645.s004.docx]

# S4 Text. Statistical Considerations

***Primary Outcomes***

The primary outcome of this study will be HIV test uptake over the previous three months. This will be assessed by self-report during follow up survey and triangulated with HIV testing surveillance data from the CDC. A difference of 10% in testing rate (assuming a proportion of HIV testing of 35% during the crowdsourced intervention period and 25% during the conventional intervention period) was chosen based on existing levels of HIV testing and what would be feasible and have public health importance in the Chinese context. The CDC measurement of the primary outcome will be the difference between HIV testing comparing the three months immediately prior to the intervention and the three months of the intervention.

***Secondary Outcomes***

A number of secondary outcomes will also be measured. These include syphilis testing, condomless sex, community engagement, testing stigma, and others (Appendix 1). Outcomes will also be stratified based on the level of engagement in developing the intervention, and based on the personal level of engagement during the stage of intervention implementation.

***Sample Size Calculation***

We used a binary outcome stepped-wedge randomized controlled trial design for sample size calculation. The required sample size is calculated for the primary outcome. To calculate sample size, we assumed that a crowdsourced intervention will be superior to a conventional method in promoting HIV testing among MSM who never tested for HIV. Assuming a proportion of HIV testing of 35% during the crowdsourced period and 25% during the conventional period, total number of clusters of eight, total number of time period for intervention of four, coefficient of variation of 0.4 (usually between 0.15 and 0.4), 2-sided alpha=0.05, 90% power, and 30% loss to follow up, then the total sample size will be 1040 men (130 for each city). To further improve the power for sub-analysis and secondary outcomes, we will increase the sample size to 1280 men (160 for each city). The calculation was made using the formulas developed by [Michael A. Hussey](http://www.sciencedirect.com.libproxy.lib.unc.edu/science/article/pii/S1551714406000632) et al. (<http://faculty.washington.edu/jphughes/pubs.html>).

| P_i_* | P_c_^$^ | Number of clusters | Number of time periods | Coefficient of variation | Alpha | Power | Loss to follow up | Sample size for each cluster | Total sample size (8 clusters) |
| --- | --- | --- | --- | --- | --- | --- | --- | --- | --- |
| 30 | 20 | 8 | 5 | 0.4 | 0.05 | 0.9 | 30% | 114 | 912 |
| 30 | 20 | 8 | 5 | 0.15 | 0.05 | 0.9 | 30% | 101 | 808 |
| 30 | 15 | 8 | 5 | 0.4 | 0.05 | 0.9 | 30% | 44 | 352 |
| 30 | 15 | 8 | 5 | 0.15 | 0.05 | 0.9 | 30% | 37 | 296 |
| **35** | **25** | **8** | **5** | **0.4** | **0.05** | **0.9** | **30%** | **130** | **1040** |
| 35 | 25 | 8 | 5 | 0.15 | 0.05 | 0.9 | 30% | 119 | 952 |
| 35 | 20 | 8 | 5 | 0.4 | 0.05 | 0.9 | 30% | 53 | 424 |
| 35 | 20 | 8 | 5 | 0.15 | 0.05 | 0.9 | 30% | 44 | 352 |
| 40 | 30 | 8 | 5 | 0.4 | 0.05 | 0.9 | 30% | 141 | 1128 |
| 40 | 30 | 8 | 5 | 0.15 | 0.05 | 0.9 | 30% | 133 | 1064 |
| 40 | 25 | 8 | 5 | 0.4 | 0.05 | 0.9 | 30% | 60 | 480 |
| 40 | 25 | 8 | 5 | 0.15 | 0.05 | 0.9 | 30% | 51 | 408 |
| 45 | 35 | 8 | 5 | 0.4 | 0.05 | 0.9 | 30% | 149 | 1192 |
| 45 | 35 | 8 | 5 | 0.15 | 0.05 | 0.9 | 30% | 141 | 1128 |
| 45 | 30 | 8 | 5 | 0.4 | 0.05 | 0.9 | 30% | 64 | 512 |
| 45 | 30 | 8 | 5 | 0.15 | 0.05 | 0.9 | 30% | 57 | 456 |

Note: *Pi: probability of HIV testing during intervention period; ^$^Pc: probability of HIV testing during control period.

**Analysis Plan**

***Primary Outcome Analyses***

The primary outcome will be self-reported HIV testing uptake in the past three months. We will examine the hypothesis comparing the superiority of the crowdsourced intervention compared to conventional HIV test uptake campaigns. In our study, since the outcome is binary, generalized linear mixed models (GLMM) and generalized estimating equations (GEE) can be used in our study for the primary outcome analysis. However, since we only have eight clusters, GLMM will be used for primary data analysis in our study; GLMM is preferred in studies with a small number of clusters. The model will include intervention status and time as fixed effects and site and individuals as random effects. The estimated intervention effects will be reported with 95% CIs and p values. Descriptive analysis will be used to summarize the characteristics and behaviours of the participants at baseline and follow-up surveys.

***Secondary Outcome Analyses***

Similar analyses will be conducted for binary secondary outcomes (continues variables will be categorized into binary variable), including frequency of syphilis testing, frequency of HIV testing (among those with previous HIV testing), condomless sex, community engagement, awareness of HIV status, empowerment and others. In addition, since four cities will implement more intensive in-person events to promote engagement (Guangzhou, Shenzhen, Qingdao, and Jinan) during intervention development, sub-analysis will be conducted to evaluate the potential effect of in-person events to promote HIV testing and other secondary outcomes among Chinese MSM. In addition, secondary analysis will investigate an interaction effect between intervention and community engagement (both for engagement during the intervention development stage and engagement during the intervention implementation stage, at personal level).

***Sub-Analyses***

Sub-analysis will also be conducted to compare the effect of the intervention in participants with different age (less than 30 versus 30 or older), and to compare two delivery methods for individual-level intervention: WeChat message vs. SMS text message.
