# Supplementary material for: Crowdsourcing to expand HIV testing among men who have sex with men in China: A closed cohort stepped wedge cluster randomized controlled trial
Source: PLoS Med. 2018 Aug 28;15(8):e1002645. doi: 10.1371/journal.pmed.1002645 (PMC6112627; doi:10.1371/journal.pmed.1002645)
Supplement: S1 Table — MSM, men who have sex with men. (DOCX) [file pmed.1002645.s008.docx]

# S1 Table HIV Testing Rate by City among Chinese MSM in China, 2016-2017 (N = 1219)

|  |  | **HIV testing proportion in the past 3 months,**  **% (no. tested/no. evaluated) *** | | | |
| --- | --- | --- | --- | --- | --- |
|  | **Enrollment, n=** | **1st follow-up** | **2nd follow up** | **3rd follow-up** | **4th follow-up** |
| Group 1. Guangzhou | 203 | 16.9 (26/154) | 37.6 (56/149) | 23.7 (37/156) | 35.1 (52/148) |
| Group 1. Yantai | 180 | 21.6 (30/139) | 32.8 (43/131) | 27.5 (33/120) | 28.4 (36/127) |
| Group 2. Jiangmen | 139 | 17.5 (21/120) | 33.6 (40/119) | 30.8 (33/107) | 37.3 (38/102) |
| Group 2. Jinan | 189 | 21.4 (34/159) | 31.9 (45/141) | 27.7 (36/130) | 35.9 (47/131) |
| Group 3. Zhuhai | 134 | 20.7 (23/111) | 27.4 (31/113) | 44.6 (49/110) | 40.6 (43/106) |
| Group 3. Qingdao | 182 | 19.2 (28/146) | 21.1 (30/142) | 54.1 (73/135) | 38.5 (50/130) |
| Group 4. Shenzhen | 203 | 20.4 (33/162) | 25.5 (41/161) | 28.1 (45/160) | 50.7 (75/148) |
| Group 4. Jining | 151 | 22.5 (29/129) | 31.8 (42/132) | 30.2 (38/126) | 46.1 (53/115) |

Shaded cells represent intervention periods on the basis of the stepped wedge design, with dark gray representing active intervention periods, light gray post-intervention periods, and white cells control periods. At enrollment, all participants reported having not tested for HIV within the past three months (required for study eligibility).

*We included 1219 participants who filled out at least one of the four follow-up surveys in this analysis.
