# Supplementary material for: Crowdsourcing to expand HIV testing among men who have sex with men in China: A closed cohort stepped wedge cluster randomized controlled trial
Source: PLoS Med. 2018 Aug 28;15(8):e1002645. doi: 10.1371/journal.pmed.1002645 (PMC6112627; doi:10.1371/journal.pmed.1002645)
Supplement: S2 Table — (DOCX) [file pmed.1002645.s009.docx]

# S2 Table. Baseline Characteristics of Study Participants Stratified by Loss to Follow-up in the Crowdsourcing Stepped Wedge Randomized Controlled Trial in China in 2016-2017

|  | **Lost to FU pre-intervention (n=122)*** | **Lost to FU during intervention (n=90)^#^** | **Completed last follow -up survey or seroconverted (n=1075)^¶^** | ***p*-value** |
| --- | --- | --- | --- | --- |
| Gender |  |  |  | 0.30 |
| Male | 113 (93%) | 84 (93%) | 1026 (95%) |  |
| Transgender | 9 (7%) | 6(7%) | 49 (5%) |  |
| Age, years |  |  |  | **0.009** |
| 16-20 | 20 (16%) | 9 (10%) | 264 (24%) |  |
| 21-25 | 43 (35%) | 32 (36%) | 363 (34%) |  |
| 26-30 | 32 (26%) | 24 (27%) | 267 (25%) |  |
| >30 | 27 (22%) | 25 (28%) | 181 (17%) |  |
| Marital status |  |  |  | 0.43 |
| Never married | 103 (84%) | 76 (84%) | 943 (88%) |  |
| Married | 19 (16%) | 14 (16%) | 132 (11%) |  |
| Annual income, US$ |  |  |  | **0.03** |
| <3000 | 16 (13%) | 11 (12%) | 244 (23%) |  |
| 3000-6000 | 32 (26%) | 18 (20%) | 237 (22%) |  |
| 6001-9500 | 30 (25%) | 27 (30%) | 336 (31%) |  |
| 9501-12500 | 27 (22%) | 26 (29%) | 164 (15%) |  |
| ≥12501 | 17 (14%) | 8 (9%) | 94 (9%) |  |
| Highest education |  |  |  | 0.73 |
| High school or below | 42 (34%) | 29 (32%) | 388 (36%) |  |
| College or beyond | 80 (66%) | 61 (68%) | 687(64%) |  |
| Sexual orientation |  |  |  | 0.58 |
| Gay | 87 (71%) | 69 (77%) | 769 (72%) |  |
| Bisexual | 35 (29%) | 21 (23%) | 306 (28%) |  |
| Disclosure of sexual orientation |  |  |  | 0.25 |
| Disclosed to others | 71 (58%) | 58 (64%) | 707 (63%) |  |
| Not disclosed to others | 51 (42%) | 32 (36%) | 368 (38%) |  |
| Ever HIV tested (from baseline)  No  Yes | 80 (66%)  42 (34%) | 46 (51%)  44 (49%) | 617 (57%)  458 (43) | 0.09 |

Data are n (%). * Lost to follow-up pre-intervention was defined as missed all the pre-intervention follow-up surveys; # Lost to follow up during the intervention periods was defined as attended all the preintervention surveys but missed the follow-up following the intervention. ^¶^72 people seroconverted during the first nine months follow-up period; ^‡^ in the past three months.
