# Supplementary material for: Crowdsourcing to expand HIV testing among men who have sex with men in China: A closed cohort stepped wedge cluster randomized controlled trial
Source: PLoS Med. 2018 Aug 28;15(8):e1002645. doi: 10.1371/journal.pmed.1002645 (PMC6112627; doi:10.1371/journal.pmed.1002645)
Supplement: S3 Table — (DOCX) [file pmed.1002645.s010.docx]

# S3 Table. Incident HIV testers in each follow-up period

|  |  | **Incident HIV testers in each follow-up period*** | | | |  |
| --- | --- | --- | --- | --- | --- | --- |
|  | **Participants who completed at least one follow-up survey** | **1st follow-up** | **2nd follow up** | **3rd follow-up** | **4th follow-up** | **Total** |
| Group 1 | 325 | 56 | 79 | 24 | 33 | 192 (59.1%) |
| Group 2 | 292 | 55 | 62 | 25 | 32 | 174 (60.0%) |
| Group 3 | 281 | 51 | 34 | 74 | 28 | 187 (66.5%) |
| Group 4 | 321 | 62 | 50 | 34 | 56 | 202 (62.9%) |
|  |  | **Incident HIV testers (among participants who had never tested for HIV at baseline) in each follow-up period^#^** | | | |  |
|  | **Participants who completed at least one follow-up survey** | **1st follow-up** | **2nd follow up** | **3rd follow-up** | **4th follow-up** | **Total** |
| Group 1 | 198 | 25 | 46 | 12 | 20 | 103 (52.0%) |
| Group 2 | 159 | 22 | 37 | 10 | 19 | 88 (55.3%) |
| Group 3 | 151 | 20 | 15 | 40 | 14 | 89 (58.9%) |
| Group 4 | 191 | 29 | 30 | 15 | 36 | 110 (57.6%) |

Shaded cells represent intervention periods on the basis of the stepped wedge design, with dark gray representing active intervention periods, light gray post-intervention periods, and white cells control periods. At enrollment, all participants reported having not tested for HIV within the past three months (required for study eligibility).

*We included 1219 participants who filled out at least one of the four follow-up surveys in this analysis; ^#^We included 699 participants who filled out at least one of the four follow-up surveys in this analysis and had never tested for HIV at baseline.
