# Supplementary material for: Crowdsourcing to expand HIV testing among men who have sex with men in China: A closed cohort stepped wedge cluster randomized controlled trial
Source: PLoS Med. 2018 Aug 28;15(8):e1002645. doi: 10.1371/journal.pmed.1002645 (PMC6112627; doi:10.1371/journal.pmed.1002645)
Supplement: S4 Table — MSM, men who have sex with men. (DOCX) [file pmed.1002645.s011.docx]

# S4 Table. HIV Testing Frequency over Four Follow-Up Periods among MSM who tested for HIV (N = 755)

| **Number of follow-up periods during which participant reported HIV testing** | **Number of participants** |
| --- | --- |
| 1 | 395 |
| 2 | 211 |
| 3 | 107 |
| 4 | 42 |
| **Total** | **755** |
