# Supplementary material for: Crowdsourcing to expand HIV testing among men who have sex with men in China: A closed cohort stepped wedge cluster randomized controlled trial
Source: PLoS Med. 2018 Aug 28;15(8):e1002645. doi: 10.1371/journal.pmed.1002645 (PMC6112627; doi:10.1371/journal.pmed.1002645)
Supplement: S5 Table — (DOCX) [file pmed.1002645.s012.docx]

# S5 Table. Concordance between Self-Reported Testing Results and HIV Self-Testing Kit Results among Participants Who Used our HIV Self-Testing Platform, 2016-2017 (n=132)

|  | **# self-testing kit results returned** | **# self-testing kit results that matched self-reported results** | **Match rate, %** |
| --- | --- | --- | --- |
| Group 1 | 10 | 9 | 90.0 |
| Group 2 | 17 | 15 | 88.2 |
| Group 3 | 48 | 46 | 95.8 |
| Group 4 | 57 | 54 | 94.7 |
| Total | 132 | 124 | 93.9 |

Group 1 represents Guangzhou and Yantai; Group 2, Jiangmen and Jinan; Group 3, Zhuhai and Qingdao; Group 4, Shenzhen and Jining.
