# Supplementary material for: Crowdsourcing to expand HIV testing among men who have sex with men in China: A closed cohort stepped wedge cluster randomized controlled trial
Source: PLoS Med. 2018 Aug 28;15(8):e1002645. doi: 10.1371/journal.pmed.1002645 (PMC6112627; doi:10.1371/journal.pmed.1002645)
Supplement: S6 Table — MSM, men who have sex with men. (DOCX) [file pmed.1002645.s013.docx]

# S6 Table. HIV Self-Testing Rates by Intervention Group over Four Follow-Up Periods among Chinese MSM, 2016-2017 (N = 1219)

|  | **HIV self-testing proportion, %*** | | | |
| --- | --- | --- | --- | --- |
|  | **1st follow-up** | **2nd follow up** | **3rd follow-up** | **4th follow-up** |
| Group 1 | 10.9¶ | 27.9 | 18.5 | 21.4 |
| Group 2 | 10.4 | 26.2 | 22.8 | 22.5 |
| Group 3 | 10.1 | 14.9 | 38.4 | 27.4 |
| Group 4 | 13.4 | 21.5 | 19.9 | 39.2 |

Dark gray cells represent intervention periods on the basis of the stepped wedge design. Light gray cells represent the post- intervention period. White represents control periods prior to intervention. Follow-ups took place at three-month intervals; ¶For group 1, the HIV self-testing intervention was provided during months 4-6; *We included 1219 participants who filled out at least one of the four follow-up surveys in this analysis.
